# The silence of the labs: Ethical oversight in metabolomics research

**DOI:** 10.1371/journal.pone.0346284

**Published:** 2026-10-07

**Authors:** Joan Badia, Maria Llambrich, Rocío López-Rubio, Horacio Saggion, Raquel Cumeras

**Affiliations:** 1 Bioinformatics and Statistics Platforms, Institut de Recerca Biomèdica Catalunya Sud (IRBCatSud), Reus, Spain; 2 Electrical, Electronic and Automatic Engineering Department, Universitat Rovira i Virgili, Tarragona, Spain; 3 Oncology Department, Institut de Recerca Biomèdica Catalunya Sud (IRBCatSud), Reus, Spain; 4 Department of Engineering, Universitat Pompeu Fabra, Barcelona, Spain; Indian Institute of Technology (IIT BHU) - Varanasi, INDIA

## Abstract

Ethical oversight and transparent reporting are essential for ensuring accountability and participant protection in biomedical research. However, despite the rapid expansion of human metabolomics, the consistency of ethical reporting in this field remains unclear. Here, we introduce MinEth, a benchmark corpus and transformer-based framework for detecting ethics-related statements in metabolomics publications. The corpus comprises 120 full-text research articles annotated for key ethical categories, including approval mentions, institutional review boards, and approval identifiers. Using this resource, we fine-tuned and evaluated several transformer architectures—BERT, RoBERTa, SciBERT, and DeBERTa-v3—for automated classification of ethical statements. Across 20 independent fine-tuning runs using identical data partitions, SciBERT achieved the highest overall ethics macro-F1 (0.614 ± 0.111) and significantly exceeded the other evaluated architectures after Holm correction. DeBERTa-v3 achieved the highest mean F1 for ethics approval identifiers (0.538 ± 0.232), although it did not significantly outperform all competing models. Annotation reliability was substantial (Cohen’s κ = 0.84), confirming the robustness of the labeling scheme. Together, these results show that transformer-based language models can identify ethics-related reporting with high sensitivity and strong ranking performance, although F1-score and precision vary across architectures, target categories, and random seeds. MinEth provides an open, reproducible resource for advancing transparency, governance, and machine-readable ethics in metabolomics research.

## Introduction

Ethical oversight and transparent reporting are foundational pillars in biomedical research: they protect participants’ rights, support public trust, and enable reproducibility and accountability. Institutional review boards or ethics committees vet research protocols to balance benefits and risks, while informed consent ensures participant autonomy and clarity about data use. Transparent reporting—naming oversight bodies, providing approval identifiers, and clarifying consent scope—is crucial so that readers, reviewers, and policy makers can evaluate the rigor and legitimacy of studies [1,2]. In omics-driven biomedical science, where the scale and complexity of data introduce elevated risks, these ethical and transparency demands acquire renewed urgency.

The growth of omics research has been exponential, and metabolomics is no exception. A 2025 internal PubMed search for “human AND (metabolomics OR metabolite)” retrieved over 200,000 records, reflecting the widespread adoption of metabolite profiling in disease, environmental, and exposome research. This scale highlights both opportunity and risk: thousands of human studies rely on biospecimens, cross-study reuse, and data sharing. At the same time, molecular signatures in metabolomic data can reveal diet, lifestyle exposures, disease states, or medication histories [3,4]. Coupled with clinical or genomic metadata, they raise re-identification risks and intensify ethical demands [5–7]. At the same time, legal and governance frameworks—such as the General Data Protection Regulation (GDPR), the “Five Safes” model, and evolving funder mandates are pushing toward machine-readable and auditable ethics documentation, not only prose statements [2,8–10].

Despite this scale and sensitivity, ethical reporting in metabolomics remains inconsistent and often opaque. Studies on biomedical research-reporting practices have repeatedly shown that reports of human-subject studies may omit or incompletely report key information such as the approving ethics board, approval identifiers, and consent details [11–13]. As summarized in Table 1, the frequency of missing ethics-approval and informed-consent reporting varies widely across biomedical fields, article types, journals, and study settings. Within metabolomics, such omissions are particularly problematic because the field still lacks consistent standardized reporting of metadata [14]. Metabolomics therefore raises not only technical challenges, but also distinct ethical and regulatory concerns [15]. For example, there is no existing corpus or benchmark system for automatically extracting ethics statements or identifiers specifically from metabolomics publications. This absence limits our ability to scale audits across the literature and detect patterns of underreporting or editorial policy compliance over time.

**Table 1:** Published studies assessing the reporting of ethics approval and informed consent across biomedical research areas. NR indicates not reported.

Reference	Field / sample	Ethics approval NOT mentioned	Informed consent NOT mentioned	Neither mentioned
[16]	600 clinical trials published before and after 1997 in five major general medical journals	31% before 1997; 18% after 1997	26% before 1997; 18% after 1997	16% before 1997; 9% after 1997
[17]	93 human-subject articles published by researchers at the Istituto Superiore di Sanità in 2001	87.1%	59.1%	NR
[12]	1,133 clinical research articles in five major medical journals, 2005–2006	3.2%	5.5%	1.3%
[18]	Human-subject biomedical research originating from Sri Lanka: 291 postgraduate theses (1985–2005), 79 international full-text original research publications (1999–2004), and 100 human-subject articles from the *Ceylon Medical Journal* (1999–2005)	66.0% in theses; 62.0% in international publications; 64.0% in *Ceylon Medical Journal*	38.8% in theses; 61.0% in international publications; 63.0% in *Ceylon Medical Journal*	NR
[19]	989 primary analyses published in nine public health journals in 2006	27%	37%	NR
[20]	401 clinical research articles from three leading European otolaryngology journals, published in 2012	49.9%	42.9%	NR
[21]	9,488 clinical trials published in 12 core Chinese nursing journals, 2013–2016	74.1%	48.2%	NR
[22]	1,284 clinical studies published in five leading international nursing journals, 2015–2017	6.3%	12.5%	NR
[23]	68 online reports and 138 archived reports of research, 2004–2012	86.8% in online reports; 100% in archived reports	57.4% in online reports; 87.7% in archived reports	NR
[24]	972 COVID-19 studies involving hospitalised adult patients	9.1%	31.4%	NR
[25]	169 empirical studies in palliative and end-of-life care journals	5%	13%	NR
[13]	480 case reports and case series sampled from PubMed, 2006–2017	51.0%	NR	NR
[26]	2,053 open-access case reports and case series indexed in PubMed and published in 2021	76%	21%	NR

*Note.* Percentages are reported as stated in the original articles. When studies reported the percentage of articles mentioning ethics approval or informed consent, the percentage not mentioning the item was calculated as 100 minus the reported percentage. Because studies used different inclusion criteria and definitions, including absent, minimal, incomplete, or formal reporting, values should be compared descriptively rather than as directly equivalent estimates.

Manual curation of ethics statements across thousands of articles is untenable: statements may be scattered across *Methods*, *Acknowledgments*, or *Supplementary Materials*, written in diverse phrasing and variable completeness. This heterogeneity makes it difficult to systematically track compliance or train reliable detection schemas. Natural Language Processing (NLP), particularly recent advances in transformer-based models, offers a scalable path forward. Transformer models are neural-network architectures that learn contextual relationships between words in a sentence, allowing them to recognize patterns that depend on surrounding text. Transformer architectures [27] revolutionized NLP by enabling self-attention and contextual embedding of tokens in both directions. BERT (Bidirectional Encoder Representations from Transformers) is a foundational model that learns deep contextual embeddings by jointly training on masked language modeling (predicting masked tokens) and next-sentence prediction tasks [28]. It has become a strong baseline for many NLP tasks, including sentence classification and entity extraction [29,30]. Subsequent models such as RoBERTa (a retrained, optimized variant removing next-sentence prediction and using more data) and DeBERTa (introducing disentangled attention) further refine performance in domain-specific tasks [31,32]. Domain-specific models like SciBERT are pretrained on scientific corpora, improving performance in scholarly text [33].

Transformer-based language models have become central to biomedical and scientific text mining. In addition to general-domain architectures, domain-adapted models have improved performance in specialized corpora: BioBERT and PubMedBERT were developed for biomedical literature mining [34,35], SciBERT for scientific articles across disciplines [33], and ClinicalBERT for clinical notes [36]. These models have been widely applied to named entity recognition, relation extraction, question answering, and sentence classification, providing an appropriate methodological foundation for automated detection of specific reporting elements in research articles.

A parallel line of work has focused on extracting structured methodological, evidentiary, or metadata information from biomedical publications. The EBM-NLP corpus introduced large-scale annotations of randomized controlled trial abstracts for PICO elements (Participants/Problem, Intervention, Comparison and Outcome), including participants, interventions, and outcomes [37]. Subsequent systems such as Trialstreamer use automated extraction to support living evidence maps of randomized trials [38], while related approaches have explored deep learning for PICO recognition and transformer-based extraction from clinical trial text [39–41]. Beyond PICO extraction, fine-tuned NLP models have also been applied to method-entity extraction and structured metadata extraction from biomedical or narrative articles [42–44]. Other tools, including RobotReviewer, address reporting quality and risk-of-bias assessment by identifying methodological features such as randomization, allocation concealment, and blinding [45–47]. These studies demonstrate the feasibility of using NLP to extract structured information from biomedical papers, but their target categories mainly concern clinical evidence, trial design, methodological reporting, metadata extraction, or bias assessment rather than ethics oversight.

Beyond the biomedical research-reporting studies summarized above (see Table 1), ethics-related reporting has also been examined in NLP and AI research. Recent work has analysed formal ethical review mentions or broader ethical concern statements, including EthiCon, a corpus of ethical concern statements from ACL Anthology papers [44,48]. However, these resources address either manual reporting audits in biomedical research or ethical concerns in AI/NLP papers, and do not provide a benchmark for sentence-level detection of ethical approval, ethics committee names, or formal approval identifiers in biomedical full-text articles.

In this context, we introduce MinEth, a benchmark and framework tailored to ethical oversight reporting in metabolomics literature. MinEth includes a manually annotated corpus of 120 full-text articles from six leading journals in metabolomics and related fields (*Metabolomics*, *Metabolites*, *Journal of Proteome Research*, *Nature Communications*, *PLOS ONE*, and *Scientific Reports*). Sentences in the corpus are labeled with ethics-related categories (e.g., ETHICAL approval mention, ETH_ID identifiers, ETH_BOARD names, consent statements), and experimental context (MATRICE, SAMPLES, NUM_SAMPLES). Using this manually curated corpus, we trained and evaluated supervised transformer-based sentence classifiers including BERT, RoBERTa, SciBERT, and DeBERTa to detect statements and their subtypes automatically, repeating each model–task experiment across 20 random seeds to quantify stochastic training variability. We evaluate performance using metrics such as precision and F1, and we analyze trends over journals, publication years, and compliance levels.

MinEth addresses a specific gap by providing, to our knowledge, the first manually curated benchmark corpus focused on ethical oversight reporting in full-text human metabolomics research articles. Unlike previous biomedical NLP resources, which mainly target biomedical entities, PICO elements, clinical trial characteristics, metadata extraction, reporting quality, or risk-of-bias assessment, MinEth focuses specifically on sentence-level detection of ethics approval statements, ethics board names, and formal approval identifiers in metabolomics publications. By bridging expert curation with state-of-the-art NLP, MinEth enables scalable audits, empowers editors and funders to monitor compliance, and supports meta-science efforts toward raising transparency standards. In short, MinEth contributes both a resource (annotated corpus) and a method (model comparisons) to the infrastructure of responsible, machine-readable science in omics.

## Materials and methods

### Ethics statement

This study was based exclusively on the analysis of information reported in publicly available, previously published scientific articles. No human participants were recruited, and no individual-level participant data, medical records, or archived biological samples were accessed. The experts who performed the manual annotation were members of the research team acting in their professional capacity as annotators and were not enrolled as research participants. Therefore, ethics committee approval and participant informed consent (written or verbal) were not applicable to this study. No minors were involved.

### Corpus selection and article inclusion criteria

The corpus comprised 120 peer-reviewed metabolomics research articles published between 2007 and 2021 across six leading journals: Journal of Proteome Research, Metabolomics, Metabolites, Nature Communications, PLOS ONE, and Scientific Reports. Twenty articles per journal were selected by random sampling from PubMed and journal archives, ensuring balanced representation of publication years and publishers. All studies explicitly addressed human metabolomics or metabolite profiling, including original research articles reporting experimental or clinical results. Review papers, editorials, and purely computational works were excluded.

### Full-text acquisition and sentence preprocessing

Articles from PLOS ONE and Metabolites were downloaded directly in XML format using the publisher’s APIs, preserving the original structure, section headers, and metadata. For journals lacking machine-readable XMLs (Nature Communications, Metabolomics), articles were converted from PDF to text using PDF-DIGEST (v1.4), while Journal of Proteome Research and Scientific Reports were processed using the Dr Inventor text-mining toolkit (v3.2) due to superior handling of embedded tables and captions. All extracted texts were normalized to UTF-8 encoding, stripped of figures, tables, and references, and segmented into sentences using spaCy’s rule-based model for scientific English (v3.6). Non-English characters, mathematical symbols, and section headings were retained for contextual training.

### Manual annotation scheme and adjudication workflow

Corpus annotation was performed using the General Architecture for Text Engineering (GATE) environment (v9.0.1). Three independent annotators, each trained in biomedical text mining and ethics reporting, annotated 56 articles each, with 32 overlapping documents to measure inter-annotator consistency. Twenty-four articles (20%) were triple-annotated to enable adjudication and reliability assessment. Annotations were manually consolidated by a senior researcher to eliminate boundary inconsistencies (e.g., trailing spaces or punctuation) and harmonize tag usage. Each sentence was labeled with one or more of the following categories:

Ethics:

- ETHICAL: explicit mention of ethical approval or informed consent;
- ETH_ID: inclusion of a formal ethics identifier (approval number, protocol code);
- ETH_BOARD: name of the approving ethics committee or institutional review board;

Experimental context:

- MATRICE: biological matrix mentioned (e.g., plasma, serum, urine, feces);
- SAMPLES: reference to sample type, origin, or collection process;
- NUM_SAMPLES: explicit indication of sample size.

Annotation categories were defined a priori to capture the minimum information required to assess traceable ethical oversight in human metabolomics studies. Ethics-related categories included: ETHICAL, for explicit statements of ethics approval, informed consent, exemption, or waiver (e.g., “The study was approved by the Institutional Review Board”); ETH_BOARD, for the name of the approving ethics committee or institutional review board (e.g., “Somerset Research and Ethics Committee”); and ETH_ID, for formal approval numbers, protocol codes, or equivalent identifiers (e.g., “LREC Protocol Number: 05/Q2202/96”). Together, these categories determine whether ethical oversight was reported, who provided it, and whether it is traceable. Additional experimental-context categories captured key study descriptors, including MATRICE (e.g., “plasma samples”), SAMPLES (e.g., “blood samples were collected”), and NUM_SAMPLES (e.g., “n = 26”).

The final annotated dataset contained 62 articles used for training and internal validation, and 54 reserved for independent testing. Sentence-level annotations were exported to tabular format (CSV) through Dr Inventor’s Text Mining Library, linking text fragments with document identifiers, section origin, and annotation type.

To minimize interpretation bias and ensure consistency, annotations were conducted independently and blindly on overlapping documents, with no access to other annotators’ labels during the initial round. Ethical statements were identified at the sentence level using predefined operational criteria: sentences were labeled ETHICAL if they explicitly referred to ethics approval, institutional review, informed consent, or approval waiver; ETH_BOARD if they mentioned an ethics committee or institutional review board; and ETH_ID if they included an approval number or protocol identifier. Annotators, with complementary expertise in biomedical text mining, metabolomics, and ethics curation, were trained using a shared guideline and a pilot set of articles to harmonize label interpretation, particularly for ambiguous cases.

The annotation task was challenging because ethics-related information was sparse and heterogeneously reported across full-text articles. Relevant statements were not restricted to a single section and could appear in Methods, Acknowledgments, ethics declarations, data-availability sections, or Supplementary Materials. In addition, several sentences contained multiple ethics-related elements, whereas others provided only partial information, such as a committee name without an approval identifier or a consent statement without explicit ethics approval. Disagreements mainly involved boundary decisions between general ethics approval mentions, named oversight bodies, and formal identifiers. These cases were reviewed during adjudication, and a senior researcher harmonized tag usage, corrected boundary inconsistencies, and produced the final consolidated annotations used for model training and evaluation.

### Inter-annotator agreement and annotation bias assessment

To evaluate annotation consistency, 32 articles were annotated by more than one expert and 24 (20%) were triple-annotated. Agreement was measured using Cohen’s κ for pairwise comparisons and Fleiss’ κ for the triple-annotated subset. These statistics quantify concordance between annotators beyond random chance, where κ=1 indicates perfect agreement and κ=0 corresponds to chance-level consistency.

Cohen’s κ was calculated using the standard formulation $\kappa =(P_{o}-P_{e})/(1-P_{e})$, where $P_{o}$ is the observed proportion of agreement between two annotators and $P_{e}$ is the agreement expected by chance from the marginal distribution of their annotations. For this analysis, annotations were converted into sentence-level binary decisions for each category, indicating whether a given sentence contained a specific annotation label or not. Pairwise Cohen’s κ was then calculated between annotators for each overlapping article and annotation category. For multi-label annotations, each label was evaluated using a one-vs-rest coding scheme, and κ values were averaged across labels and annotator pairs. Fleiss’ κ was calculated using the standard formulation $\kappa =(\bar{P}-{\bar{P}}_{e})/(1-{\bar{P}}_{e})$, where $\bar{P}$ represents the mean observed agreement across annotated units and ${\bar{P}}_{e}$ represents the agreement expected by chance from the marginal distribution of category assignments. For each annotated unit *i*, observed agreement was computed as $P_{i}=[1/n(n-1)]\sum_{j=1}^{k}n_{ij}(n_{ij}-1)$, where *n* is the number of annotators, *k* is the number of possible categories, and $n_{ij}$ is the number of annotators assigning unit *i* to category *j*. In this study, Fleiss’ κ was computed at article–category level using presence/absence decisions for each annotation label across the three annotators, rather than requiring exact span-boundary overlap. This approach was used because relevant information could appear in multiple locations within the same article, and annotators could therefore select different but semantically related spans.

### Model architecture and supervised training strategy

Sentence classification was formulated as multiple sentence-level classification tasks, one for each annotation category. Transformer-based language models were fine-tuned using the Hugging Face Transformers library (v4.34). The following pretrained models were evaluated: BERT-base-uncased (Devlin et al., 2019), RoBERTa-base (Liu et al., 2019), SciBERT-base-uncased (Beltagy et al., 2019), DeBERTa-v3-base (He et al., 2021) and RoBERTa-large (Liu et al., 2019).

The annotated data were split into training/internal-validation and independent evaluation datasets. Each model was fine-tuned for four training epochs with early stopping (patience = 5) based on precision–recall (PR) AUC on the evaluation dataset, using a batch size of 16, a maximum sequence length of 200 tokens, and a learning rate of 1 × 10^−4^ optimized through AdamW. Class imbalance was mitigated by oversampling minority categories. The corpus was tokenized using the model-specific tokenizer. To assess variability associated with stochastic fine-tuning, each model–task combination was independently trained and evaluated 20 times using different random seeds (123, 234, 345, 456, 567, and 1000–1014). The held-out test partition, preprocessing procedures, optimization settings, and hyperparameters were identical across runs, while data shuffling and the internal training/validation split were controlled by the random seed. All 20 runs were retained, including runs showing marked performance degradation, because inspection confirmed that these represented genuine optimization instability rather than technical execution errors.

### Evaluation metrics and model interpretation

Performance was assessed on the held-out test set, and multiple performance metrics were calculated, including Accuracy, F1-score, Precision, Recall/Sensitivity, Specificity, ROC AUC, PR AUC, MCC and BER. Additionally, performance on the evaluation dataset was reported to visualize learning convergence and early stopping behavior. Macro-averaged metrics were reported across categories. Because ethics-related sentences represented less than 1% of the corpus, model interpretation emphasized F1-score, precision, recall, PR AUC, MCC, and BER rather than accuracy alone. Accuracy can be inflated in highly imbalanced datasets because a model may achieve high overall correctness by primarily predicting the majority non-ethics class. PR AUC is particularly informative in this setting because it focuses on the trade-off between precision and recall for rare positive classes, whereas ROC AUC may appear high even when positive examples are sparse. MCC is also well suited to imbalanced classification because it incorporates all four elements of the confusion matrix and produces a high value only when the model performs well on both the positive and negative classes

For clarity, the main evaluation metrics are briefly defined here. Accuracy measures the overall proportion of correctly classified sentences. Precision, also referred to as positive predictive value (PPV), indicates the proportion of sentences predicted as positive by the model that were correct. Recall, also referred to as sensitivity or true positive rate (TPR), indicates the proportion of true positive sentences that were detected by the model. The F1-score is the harmonic mean of precision and recall and is useful when both false positives and false negatives are relevant. Specificity, also referred to as true negative rate (TNR), measures the proportion of negative sentences correctly classified as negative. Negative predictive value (NPV) indicates the proportion of sentences predicted as negative that were truly negative. ROC AUC summarizes the ability of the model to discriminate between positive and negative sentences across classification thresholds. PR AUC summarizes the precision–recall trade-off and is particularly informative when positive examples are rare. The Matthews correlation coefficient (MCC) measures the correlation between the predicted and true class labels while accounting for true positives, true negatives, false positives, and false negatives. MCC ranges from −1–1, where 1 indicates perfect classification, 0 indicates no correlation between predicted and true labels, and −1 indicates complete disagreement between predictions and true labels. Balanced error rate (BER) was calculated as the average of the false-negative rate and false-positive rate, providing an error measure that accounts for both missed positive cases and incorrectly labelled negative cases. Lower BER values indicate better balanced classification performance. For each binary classification task, F1-score refers to the positive-class F1-score. For each reported metric, the overall ethics macro-average was calculated separately within each run as the arithmetic mean of the corresponding category-level scores for ETHICAL, ETH_BOARD, and ETH_ID. Experimental-context macro-averages were calculated analogously across MATRICE, SAMPLES, and NUM_SAMPLES. Repeated-run summary values are reported as mean ± standard deviation across the 20 random seeds.

### Statistical analysis of model comparisons

Statistical comparisons were based on the held-out test-set positive-class F1-scores obtained from the 20 repeated fine-tuning runs. Because the same random seeds and data partitions were used across architectures, seeds were treated as paired blocks. Differences among the five architectures were first assessed using the non-parametric Friedman test. When the omnibus comparison was significant, two-sided paired Wilcoxon signed-rank tests were performed for all ten model pairs, and Holm’s step-down procedure was applied within each family of comparisons to control for multiple testing. Statistical significance was defined as an adjusted *p* < 0.05. Kendall’s *W* was reported as the omnibus effect-size measure, and matched-pairs rank-biserial correlations were calculated for pairwise comparisons. For each pairwise comparison, the mean paired F1 difference was calculated as Model A minus Model B; positive values therefore favor Model A, and the sign of the rank-biserial correlation follows the same direction. Analyses were performed in Python 3.13.5 using SciPy 1.17.0.

## Results

To guide interpretation of the results, sentence-level classification refers here to the task of assigning one or more predefined labels to each sentence, such as whether it contains an ethics approval statement, the name of an ethics board, or a formal approval identifier. All transformer models were trained in a supervised setting, meaning that they learned from the adjudicated manual annotations used as gold-standard labels.

### Corpus composition

The final MinEth corpus comprised 120 peer-reviewed metabolomics research articles published between 2007 and 2021, containing 32,798 sentences after preprocessing and segmentation. Sixty-two articles (13,250 sentences) were allocated to model training and validation, and fifty-four articles (19,548 sentences) to independent evaluation. After sentence segmentation, ethical-reporting categories were extremely sparse, representing less than 1% of all sentences in the corpus.

The distribution of ethical annotations across the ETHICAL, ETH_BOARD, and ETH_ID categories is summarized in Table 2. Only a small fraction of sentences explicitly mentioned ethical approval, the responsible committee, or an approval identifier. Additional co-occurrence patterns between these categories, such as combined mentions of ethical statements and institutional boards, are shown in Table 3. Together, these distributions demonstrate that ethical-oversight statements are sparse and heterogeneous across articles, often condensed into a single sentence that integrates multiple identifiers. This pronounced imbalance justified the application of focal-loss optimization and oversampling during model training.

**Table 2:** Distribution of annotated sentences in the MinEth corpus. Counts and percentages are shown for training, validation, and test subsets.

Category	Model training (62 articles, *N* = 13,250)		Model evaluation (54 articles, *N* = 19,548)	
	Training data	Validation data	Test data	Total (%)
*Ethical Sentence*	43 (0.48%)	22 (0.50%)	63 (0.32%)	128 (0.37%)
*Ethical Board*	34 (0.38%)	18 (0.41%)	23 (0.12%)	75 (0.22%)
*Ethical ID*	16 (0.18%)	8 (0.18%)	17 (0.09%)	41 (0.12%)
*Other sentences*	8,781 (98.95%)	4,324 (98.90%)	19,445 (99.47%)	32,550 (99.24%)

**Table 3:** Co-occurrence patterns among ethics-related annotations in the MinEth corpus.

Category	Training	Test
*OVERLAP ETHICAL + BOARD*	33	32 (0.16%)
*OVERLAP ETHICAL + ID*	5	0 (0%)
*OVERLAP ETHICAL + BOARD + ID*	19	17 (0.09%)
*ONLY ETHICAL*	8	14 (0.07%)

### Annotation reliability

Inter-annotator agreement analyses confirmed substantial consistency across annotators. The average Cohen’s κ for binary tags (ETHICAL, ETH_BOARD) was 0.84, while multi-label categories achieved 0.79, indicating near-perfect reliability. In the triple-annotated subset, agreement was perfect for the ETHICAL category alone (Fleiss’ κ=1.00), indicating that explicit ethics statements were consistently identified. Lower agreement for secondary and experimental-context categories (Fleiss’ κ=0.42−0.60) reflected the fact that relevant information was often distributed across different sections of the articles, including abstracts, methods, sample collection descriptions, and ethics statements. As a result, annotators sometimes selected different but semantically related spans, or differed in whether board names, identifiers, sample counts, or matrices were annotated as independent entities. The final corpus was reviewed and harmonized before model training to correct boundary mismatches and ensure consistent use of annotation labels.

### Model performance for ethics in metabolomics

Given the strong class imbalance, with ethics-related sentences representing less than 1% of the corpus, model performance was interpreted using precision, positive-class F1-score, PR AUC, and balanced error rate (BER) in addition to accuracy. Accuracy alone may overestimate performance when most sentences belong to the non-ethics class. PR AUC is informative for rare positive classes, whereas BER summarizes error while accounting for both positive and negative cases.

To quantify stochastic fine-tuning variability, all model–task combinations were evaluated across 20 random seeds. At the macro level, SciBERT achieved the highest overall ethics macro-F1 (0.614 ± 0.111), followed by RoBERTa-large (0.503 ± 0.129), BERT-base (0.503 ± 0.108), RoBERTa-base (0.456 ± 0.085), and DeBERTa-v3 (0.453 ± 0.134) (Table 4). The Friedman test identified a significant difference among architectures ($\chi^{2}(4)=22.60$, *p* < 0.001, Kendall’s *W* = 0.283). Holm-adjusted paired Wilcoxon tests showed that SciBERT achieved significantly higher overall ethics macro-F1 than BERT-base ($p_{adj}=0.022$), DeBERTa-v3 ($p_{adj}=0.004$), RoBERTa-base ($p_{adj}=0.001$), and RoBERTa-large ($p_{adj}=0.022$). The corresponding mean paired F1 advantages for SciBERT ranged from 0.110 to 0.160, with large matched-pairs rank-biserial effect sizes ($|r_{rb}|=0.733$–0.895; S3 Table). These results support SciBERT as the strongest overall architecture across the three core ethics-related categories.

**Table 4:** Repeated-run performance of transformer architectures for ethics-related sentence classification. Values are mean ± standard deviation across 20 random-seed runs. Macro metrics were calculated separately within each run across ETHICAL, ETH_BOARD, and ETH_ID. Bold values indicate the best mean performance per metric and do not necessarily indicate statistically significant pairwise differences. Lower BER values indicate better performance.

Metric	BERT-base	DeBERTa-v3	RoBERTa-base	RoBERTa-large	SciBERT
Macro-F1	0.503 ± 0.108	0.453 ± 0.134	0.456 ± 0.085	0.503 ± 0.129	**0.614 ± 0.111**
Macro-Accuracy	0.993 ± 0.004	0.960 ± 0.095	0.992 ± 0.005	0.994 ± 0.005	**0.997 ± 0.003**
Macro-Precision	0.389 ± 0.122	0.338 ± 0.125	0.328 ± 0.078	0.367 ± 0.123	**0.497 ± 0.134**
Macro-PR AUC	0.817 ± 0.055	0.848 ± 0.071	0.859 ± 0.040	0.831 ± 0.057	**0.880 ± 0.044**
Macro-MCC	0.565 ± 0.085	0.525 ± 0.117	0.533 ± 0.069	0.574 ± 0.103	**0.659 ± 0.089**
Macro-BER	0.034 ± 0.021	0.038 ± 0.048	0.017 ± 0.007	**0.012 ± 0.009**	0.029 ± 0.027

Category-level F1-scores also varied substantially across architectures and random seeds (Table 5; Fig 1). SciBERT achieved the highest mean F1 for ETHICAL (0.707 ± 0.112) and ETH_BOARD (0.640 ± 0.195), whereas DeBERTa-v3 achieved the highest mean F1 for ETH_ID (0.538 ± 0.232). Significant omnibus differences among architectures were observed for ETHICAL ($\chi^{2}(4)=15.40$, *p* = 0.0039, Kendall’s *W* = 0.193), ETH_BOARD ($\chi^{2}(4)=9.89$, *p* = 0.042, Kendall’s *W* = 0.124), and ETH_ID ($\chi^{2}(4)=10.52$, *p* = 0.033, Kendall’s *W* = 0.131). Complete post hoc comparisons are reported in S3 Table.

**Table 5:** Category-level test-set F1-scores across repeated fine-tuning runs. Values are mean ± standard deviation across 20 random seeds. Overall ethics macro-F1 was calculated within each run across ETHICAL, ETH_BOARD, and ETH_ID. Bold values indicate the highest mean in each column and do not necessarily indicate statistically significant pairwise differences.

Model	ETHICAL	ETH_BOARD	ETH_ID	Overall ethics macro-F1
BERT-base	0.618 ± 0.205	0.548 ± 0.216	0.343 ± 0.211	0.503 ± 0.108
DeBERTa-v3	0.494 ± 0.230	0.328 ± 0.231	**0.538 ± 0.232**	0.453 ± 0.134
RoBERTa-base	0.494 ± 0.200	0.547 ± 0.229	0.327 ± 0.165	0.456 ± 0.085
RoBERTa-large	0.556 ± 0.205	0.523 ± 0.238	0.430 ± 0.167	0.503 ± 0.129
SciBERT	**0.707 ± 0.112**	**0.640 ± 0.195**	0.494 ± 0.214	**0.614 ± 0.111**

**Fig 1:**
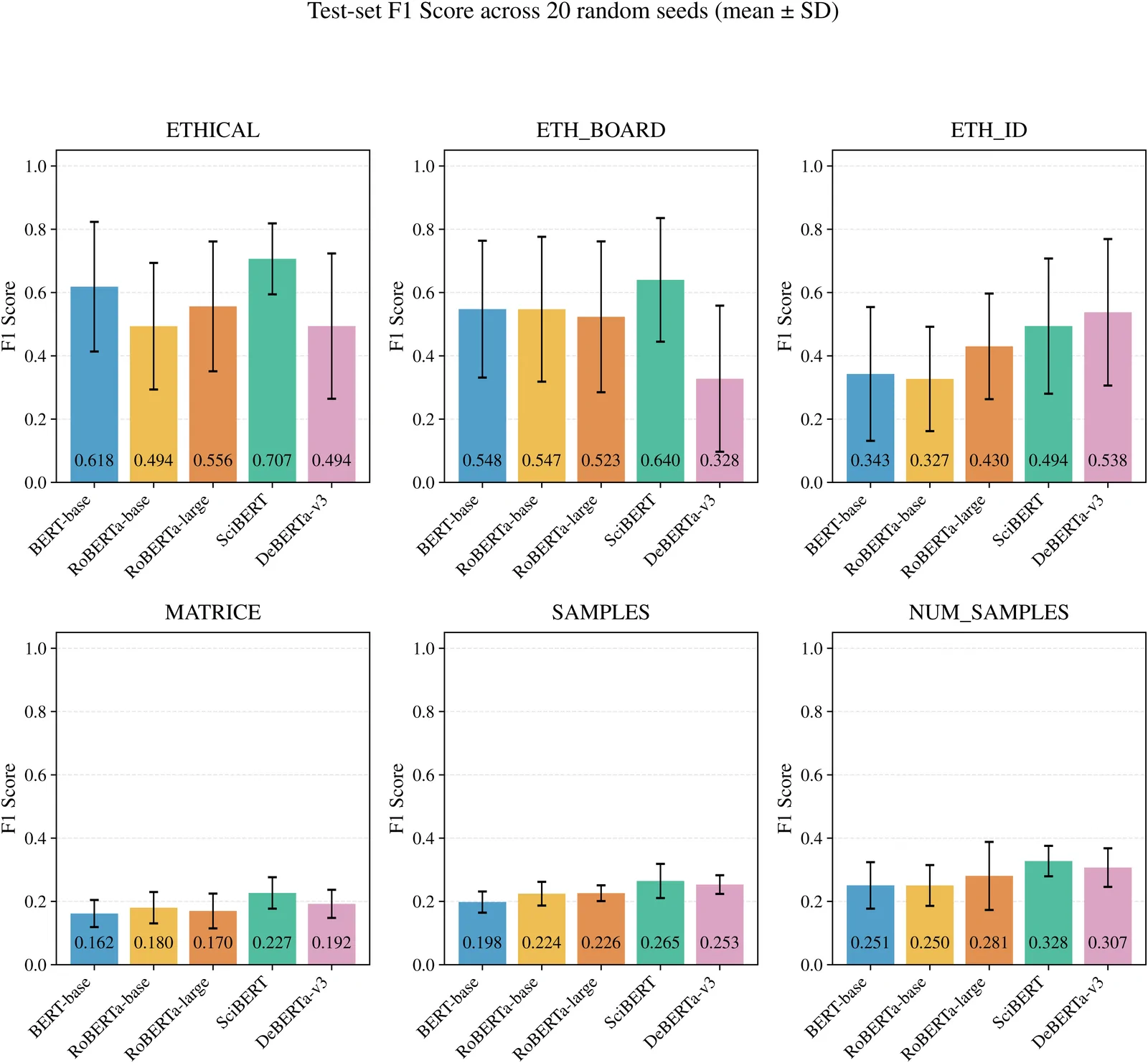
Test-set F1-scores across repeated fine-tuning runs. Bars show the mean positive-class F1-score for five transformer architectures across the ETHICAL, ETH_BOARD, ETH_ID, MATRICE, NUM_SAMPLES, and SAMPLES classification tasks. Each model–task combination was independently trained and evaluated using 20 random seeds while maintaining identical training, internal-validation, and held-out test partitions and hyperparameter settings. Error bars represent ±1 standard deviation across runs. Statistical comparisons used Friedman tests followed by paired Wilcoxon signed-rank tests with Holm correction when the omnibus test was significant.

For ETH_ID, DeBERTa-v3 achieved the highest mean F1, but it did not significantly outperform all competing architectures. After Holm correction, DeBERTa-v3 significantly exceeded RoBERTa-base ($p_{adj}=0.020$; mean paired difference = 0.211; $r_{rb}=0.752$), but not BERT-base ($p_{adj}=0.109$), RoBERTa-large ($p_{adj}=0.768$), or SciBERT ($p_{adj}=0.777$). Its result should therefore be interpreted as the highest average ETH_ID F1 rather than as consistent superiority over all alternatives.

The error bars in Fig 1 show appreciable seed-to-seed variability for several sparse ethics categories. In particular, marked degradation in a small number of DeBERTa-v3 ETH_BOARD runs increased the corresponding standard deviation. These runs were retained because they reflected genuine training instability rather than technical failure. Complete category-level performance metrics are provided in S1 Table. Omnibus Friedman-test results are reported in S2 Table, and Holm-adjusted pairwise Wilcoxon signed-rank comparisons are provided in S3 Table. Complete per-tasks figure metrics are presented in the following order: accuracy (S1 Fig), F1-score (Fig 1), precision (S2 Fig), recall (S3 Fig), ROC AUC (S4 Fig), PR AUC (S5 Fig), MCC (S6 Fig), and BER (S7 Fig).

### Model performance for samples in metabolomics

Performance for the experimental-context categories remained substantially lower than for the core ethics-related categories (Table 6; Fig 1). Across MATRICE, SAMPLES, and NUM_SAMPLES, SciBERT achieved the highest mean macro-F1 (0.273 ± 0.034) and macro-PR AUC (0.226 ± 0.025), followed by DeBERTa-v3 for macro-F1 (0.251 ± 0.028). The Friedman test showed a significant overall difference among architectures ($\chi^{2}(4)=36.08$, *p* < 0.001, Kendall’s *W* = 0.451). After Holm correction, SciBERT achieved significantly higher macro-F1 than BERT-base ($p_{adj}<0.001$), RoBERTa-base ($p_{adj}=0.002$), and RoBERTa-large ($p_{adj}=0.014$), but not DeBERTa-v3 ($p_{adj}=0.120$). For the significant comparisons, the mean paired F1 advantages for SciBERT were 0.070 versus BERT-base, 0.055 versus RoBERTa-base, and 0.048 versus RoBERTa-large, with $\left|r_{rb}\right|$ values of 0.990, 0.857, and 0.752, respectively (S3 Table). These results identify SciBERT and DeBERTa-v3 as the strongest average performers for experimental-context extraction, while showing that performance remained modest and variable across tasks.

**Table 6:** Repeated-run performance of transformer architectures for experimental-context sentence classification. Values are mean ± standard deviation across 20 random-seed runs. Macro metrics were calculated separately within each run across MATRICE, SAMPLES, and NUM_SAMPLES. Bold values indicate the best mean performance per metric and do not necessarily indicate statistically significant pairwise differences. Lower BER values indicate better performance.

Metric	BERT-base	DeBERTa-v3	RoBERTa-base	RoBERTa-large	SciBERT
Macro-F1	0.203 ± 0.024	0.251 ± 0.028	0.218 ± 0.031	0.225 ± 0.041	**0.273 ± 0.034**
Macro-Accuracy	0.981 ± 0.008	**0.984 ± 0.006**	0.980 ± 0.008	0.965 ± 0.072	0.983 ± 0.008
Macro-Precision	0.171 ± 0.035	0.191 ± 0.036	0.160 ± 0.037	0.164 ± 0.035	**0.212 ± 0.052**
Macro-PR AUC	0.134 ± 0.020	0.174 ± 0.014	0.180 ± 0.024	0.167 ± 0.022	**0.226 ± 0.025**
Macro-MCC	0.226 ± 0.020	0.277 ± 0.017	0.253 ± 0.019	0.255 ± 0.043	**0.304 ± 0.025**
Macro-BER	0.318 ± 0.025	0.277 ± 0.032	0.270 ± 0.034	0.279 ± 0.041	**0.255 ± 0.038**

At the individual-task level, SciBERT achieved the highest mean F1 for MATRICE (0.227 ± 0.050), SAMPLES (0.265 ± 0.054), and NUM_SAMPLES (0.328 ± 0.048). However, several pairwise differences were not significant after correction, including the aggregate comparison between SciBERT and DeBERTa-v3. Consequently, individual-task rankings are interpreted descriptively rather than as evidence of universal model superiority. The broad semantic variability, sparse occurrence, and frequent dependence on article-level context likely contribute to the lower performance observed for these auxiliary categories.

### Article-level ethics-reporting completeness across journals and years

Finally, we assessed article-level patterns in ethics reporting across the 120 articles in the MinEth corpus. Overall, 83 articles (69.2%) reported ethics-related information, 70 (58.3%) reported an ethics committee or institutional review board, and 37 (30.8%) reported a formal approval identifier. Complete ethics reporting, defined as the presence of all three elements, was observed in 37 articles (30.8%), whereas 37 articles (30.8%) contained no reported ethics-related information.

Ethics-reporting completeness varied across journals, indicating heterogeneity in reporting practices (Fig 2). Temporal analysis suggested that approval identifiers and complete reporting were more frequent in recent years, particularly from 2018 onwards. When grouped into publication periods, general ethics-related reporting remained relatively stable, with ETHICAL annotations present in 66.7% of articles published in 2007–2013, 67.6% in 2014–2017, and 70.4% in 2018–2021. Ethics committee or institutional review board reporting also remained similar across periods (60.0%, 55.9%, and 59.2%, respectively). In contrast, formal approval identifiers were more frequently reported in the most recent period, increasing from 26.7% in 2007–2013 and 14.7% in 2014–2017 to 39.4% in 2018–2021. Complete ethics reporting followed the same pattern, reaching 39.4% in 2018–2021 compared with 26.7% and 14.7% in the earlier periods. Articles with no reported ethics-related information persisted across all periods at approximately 30%.

**Fig 2:**
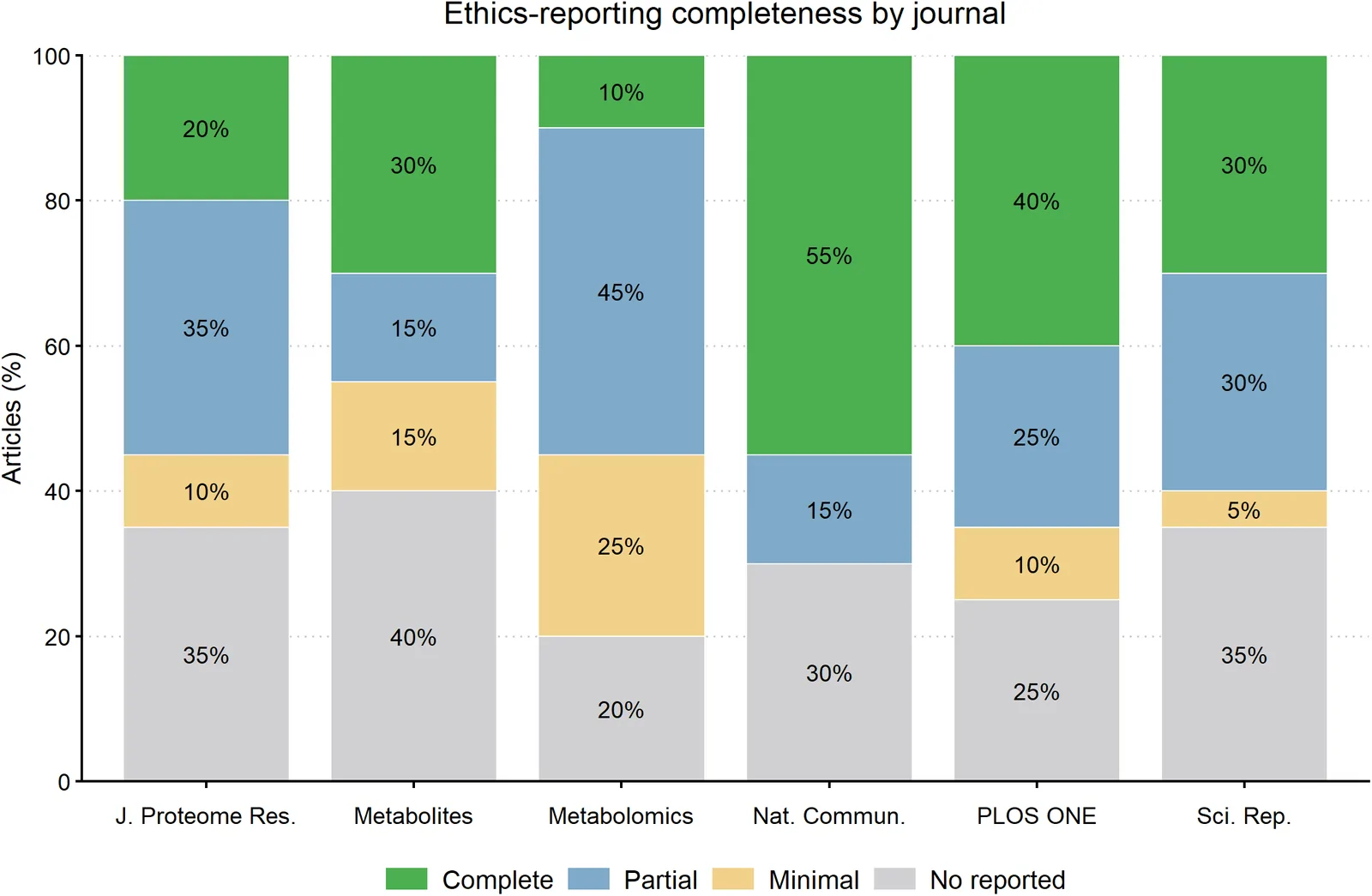
Ethics-reporting completeness by journal in the MinEth corpus. Articles were classified according to the presence of ETHICAL, ETH_BOARD, and ETH_ID annotations. Complete reporting indicates the presence of all three elements; partial reporting indicates an ethics-related statement together with either an ethics board mention or an approval identifier; minimal reporting indicates an ethics statement only; and no reported ethics information indicates absence of all three elements.

## Discussion

The systematic analysis of ethical reporting in metabolomics reveals both progress and persistent variability in how researchers communicate oversight and consent. By benchmarking transformer-based models across repeated fine-tuning runs, this study shows that ethics-related sentence-classification performance depends on both model architecture and target category. SciBERT achieved the highest overall ethics macro-F1 and significantly exceeded the other evaluated architectures across 20 random seeds. This result is consistent with a benefit from pretraining on scientific text for the present corpus, although architecture and pretraining domain cannot be fully disentangled in the current comparison. At the category level, model rankings were less uniform: DeBERTa-v3 achieved the highest mean F1 for ETH_ID, but did not significantly outperform all competing models after multiple-comparison correction. These findings demonstrate the importance of reporting repeated-run variability rather than relying on a single fine-tuning run.

Article-level analysis confirms that incomplete ethics reporting remains common in human metabolomics publications. Although 69.2% of articles included some ethics-related information, only 30.8% reported all elements required for traceable oversight: an ethics statement, identification of the responsible ethics committee or institutional review board, and a formal approval identifier. Conversely, 30.8% of articles contained no ethics-related information detectable by the MinEth annotation scheme. This proportion falls within the broad range reported in prior biomedical meta-research. For instance, missing ethics-approval reporting has been observed in 27–31% of public health and clinical trial publications, but is substantially higher in other contexts such as biomedical studies from Sri Lanka, Chinese nursing trials, case reports, and case series, where it ranges from approximately 51% to 76% [13,16,18,19,21,26]. By contrast, journal-specific analyses report much lower rates, including 3.2% in major medical journals, 6.3% in leading international nursing journals, 9.1% in COVID-19 studies, and 5% in palliative and end-of-life care journals [12,22,24,25]. Together, these comparisons position metabolomics in an intermediate range, neither among the poorest-performing areas nor aligned with the most stringent reporting environments. Importantly, MinEth extends prior work by assessing not only the presence of ethics approval but also the reporting of the oversight body and formal approval identifier, both essential for traceability. The low prevalence of approval identifiers in this corpus highlights a persistent gap in traceable ethics reporting within metabolomics.

Manual annotation and automated detection played complementary roles in this study. Manual annotation in GATE provided the expert-derived reference standard used to train and evaluate the supervised transformer classifiers. This process offered high interpretability and reliability, as reflected by the inter-annotator agreement, but required expert review of full-text articles and remained difficult to scale. This limitation was especially relevant because ethics-related statements represented less than 1% of all sentences and appeared in heterogeneous locations across articles, including Methods, Acknowledgments, ethics declarations, and Supplementary Materials. In contrast, once trained on the adjudicated corpus, transformer-based sentence classifiers enabled rapid and reproducible screening of large sentence collections. Thus, MinEth does not replace manual expert assessment; rather, it converts manual annotation into a reusable benchmark that supports scalable detection of ethics-related reporting across the literature.

The practical utility of the framework should also be interpreted in light of the lower performance observed for the experimental-context categories. Although the models achieved stronger performance for the core ethics-related categories, performance for experimental-context classification remained modest, with the highest mean macro-F1 reaching 0.273 ± 0.034 across repeated runs. Although SciBERT achieved the highest average performance, its macro-F1 was not significantly different from that of DeBERTa-v3 after multiple-comparison correction. This indicates that the current framework is more reliable for detecting ethics approval statements, ethics board mentions, and approval identifiers than for extracting sample matrices, sample descriptions, or sample-size information. The lower performance of these auxiliary categories likely reflects their broader semantic variability, sparse occurrence, and frequent dependence on article-level context rather than isolated sentence-level cues. Therefore, in its current form, MinEth should primarily be considered a framework for ethics-reporting detection, whereas experimental-context extraction should be interpreted as preliminary and requiring further methodological development.

Despite these advances, several limitations remain. The corpus reflects metabolomics publications only, and ethical reporting conventions may differ across omics disciplines or journals. Although annotation consistency was high (Cohen’s κ=0.84), some annotation uncertainty and potential annotation bias remain. Relevant ethics-related information was sparse and often distributed across different sections of the article, which could lead annotators to select different but semantically related spans. In addition, although annotators were trained using shared guidelines, this common training and domain expertise may have introduced interpretation bias toward similar readings of ambiguous statements. The use of independent blind annotation, predefined operational criteria, and subsequent adjudication was intended to minimize these effects, but cannot completely eliminate curator-dependent decisions. Moreover, the current annotation schema was designed to capture the core elements required for traceable ethics reporting, namely whether ethical oversight was reported, which committee or institutional review board provided oversight, and whether a formal approval identifier was available. However, these categories are not intended to exhaustively cover all possible ethics-governance information. For example, detailed consent scope, data-sharing permissions, privacy safeguards, participant withdrawal rights, and secondary-use restrictions were not annotated as separate categories in the present version of MinEth. Although the repeated-run analysis quantified variability arising from stochastic fine-tuning, all 20 runs used the same training, internal-validation, and held-out test partitions. The statistical comparisons therefore capture optimization-related variability but do not incorporate uncertainty associated with alternative corpus samples or data splits. The marked degradation observed in a small number of runs was retained as genuine training instability and indicates that individual fine-tuning outcomes can be sensitive to initialization, particularly for the sparsest categories. Future evaluation should incorporate repeated cross-validation and external validation on independently collected corpora. Further work should expand MinEth to include additional categories, such as informed consent language or data-sharing statements, and evaluate model generalization on unseen corpora.

Beyond metabolomics, MinEth could support broader biomedical research governance by helping editors, reviewers, funders, and institutions identify articles in which ethics-related information is missing, incomplete, or difficult to locate. This includes the absence of reported ethics committee or institutional review board approval, consent statements, and approval identifiers. Similar models could also enrich repository metadata by making ethical oversight information more searchable and machine-readable. These applications should be viewed as decision-support tools for scalable monitoring, not as replacements for human ethical review.

## Conclusions

This study establishes MinEth as the first benchmark dedicated to the automated detection of ethical oversight in metabolomics publications. Across 20 repeated fine-tuning runs, SciBERT achieved the strongest overall performance across ETHICAL, ETH_BOARD, and ETH_ID, whereas DeBERTa-v3 achieved the highest mean F1 for ethics approval identifiers without consistently outperforming all other architectures. The repeated-run analysis demonstrates that performance is influenced by both target category and stochastic training variability. The models generally achieved high sensitivity and strong PR AUC for the core ethics-related categories, although precision and F1-score remained variable, particularly for sparse identifiers and experimental-context information. The annotated corpus, open-source code, and fine-tuned model checkpoints together provide a reproducible framework for large-scale auditing of research transparency. Beyond metabolomics, MinEth illustrates how computational methods can support editors, funders, and institutions in monitoring ethical compliance and fostering responsible, machine-readable reporting practices in omics-driven biomedical science.

## Supporting information

S1 Fig Test-set accuracy across repeated fine-tuning runs. Mean accuracy and standard deviation across 20 random seeds are shown for each transformer architecture and classification task.
(EPS)

S2 Fig Test-set precision across repeated fine-tuning runs. Mean positive-class precision and standard deviation across 20 random seeds are shown for each transformer architecture and classification task.
(EPS)

S3 Fig Test-set recall across repeated fine-tuning runs. Mean positive-class recall and standard deviation across 20 random seeds are shown for each transformer architecture and classification task.
(EPS)

S4 Fig Test-set ROC AUC across repeated fine-tuning runs. Mean ROC AUC and standard deviation across 20 random seeds are shown for each transformer architecture and classification task.
(EPS)

S5 Fig Test-set PR AUC across repeated fine-tuning runs. Mean PR AUC and standard deviation across 20 random seeds are shown for each transformer architecture and classification task.
(EPS)

S6 Fig Test-set Matthews correlation coefficient across repeated fine-tuning runs. Mean MCC and standard deviation across 20 random seeds are shown for each transformer architecture and classification task.
(EPS)

S7 Fig Test-set balanced error rate across repeated fine-tuning runs. Mean BER and standard deviation across 20 random seeds are shown for each transformer architecture and classification task. Lower BER values indicate better performance.
(EPS)

S1 Table Category-level test-set performance across repeated fine-tuning runs. Mean and standard deviation across 20 random seeds are reported for the evaluated metrics and classification tasks.
(PDF)

S2 Table Omnibus statistical comparisons among model architectures. Friedman test statistics, degrees of freedom, *p*-values, and Kendall’s *W* are reported for the overall ethics macro-F1, experimental-context macro-F1, and individual classification tasks.
(PDF)

S3 Table Pairwise model comparisons across repeated runs. Paired Wilcoxon signed-rank test results include unadjusted and Holm-adjusted *p*-values, mean paired F1 differences (Model A minus Model B), and matched-pairs rank-biserial correlations.
(PDF)

S1 Data Per-seed model-performance metrics. Complete test, internal-validation, and training metrics for the 20 random-seed runs. In the data file, the task label SAMPLE corresponds to the SAMPLES category used in the manuscript.
(CSV)
