# Supplementary material for: The silence of the labs: Ethical oversight in metabolomics research
Source: PLoS One. 2026 Oct 7;21(10):e0346284. doi: 10.1371/journal.pone.0346284 (PMC13645098; doi:10.1371/journal.pone.0346284)
Supplement: Supplementary file 1 [file pone.0346284.s008.pdf]

Table S1: Category-level test-set performance across repeated fine-tuning runs. Values are mean ± standard deviation across 20 random seeds. F1-score, precision, and recall refer to the positive class. Bold values indicate the best mean performance for each metric within each classification task. Lower BER values indicate better performance.

Metric	BERT-base	DeBERTa-v3	RoBERTa-base	RoBERTa-large	SciBERT
**A. ETHICAL**					
Accuracy	0.994 ± 0.006	0.990 ± 0.010	0.991 ± 0.009	0.991 ± 0.012	**0.997 ± 0.002**
F1-score	0.618 ± 0.205	0.494 ± 0.230	0.494 ± 0.200	0.556 ± 0.205	**0.707 ± 0.112**
Precision	0.506 ± 0.233	0.368 ± 0.223	0.360 ± 0.193	0.419 ± 0.198	**0.589 ± 0.167**
Recall	0.923 ± 0.042	0.960 ± 0.023	0.956 ± 0.022	**0.967 ± 0.014**	0.937 ± 0.050
ROC AUC	0.989 ± 0.005	0.992 ± 0.008	**0.995 ± 0.004**	0.991 ± 0.007	0.992 ± 0.006
PR AUC	0.871 ± 0.043	0.887 ± 0.106	0.902 ± 0.050	0.892 ± 0.030	**0.909 ± 0.038**
MCC	0.659 ± 0.165	0.563 ± 0.184	0.562 ± 0.159	0.613 ± 0.168	**0.732 ± 0.092**
BER	0.041 ± 0.020	0.025 ± 0.010	0.026 ± 0.009	**0.021 ± 0.007**	0.033 ± 0.024
**B. ETH_BOARD**					
Accuracy	0.994 ± 0.008	0.893 ± 0.284	0.991 ± 0.015	0.993 ± 0.007	**0.996 ± 0.004**
F1-score	0.548 ± 0.216	0.328 ± 0.231	0.547 ± 0.229	0.523 ± 0.238	**0.640 ± 0.195**
Precision	0.421 ± 0.210	0.226 ± 0.205	0.413 ± 0.213	0.389 ± 0.228	**0.517 ± 0.216**
Recall	0.934 ± 0.056	0.973 ± 0.027	0.980 ± 0.021	**0.999 ± 0.005**	0.963 ± 0.056
ROC AUC	0.998 ± 0.002	0.998 ± 0.003	0.999 ± 0.002	**0.999 ± 0.001**	0.999 ± 0.005
PR AUC	0.789 ± 0.085	0.869 ± 0.105	0.870 ± 0.057	0.855 ± 0.082	**0.893 ± 0.049**
MCC	0.601 ± 0.174	0.413 ± 0.217	0.607 ± 0.188	0.594 ± 0.192	**0.683 ± 0.154**
BER	0.036 ± 0.027	0.067 ± 0.138	0.015 ± 0.011	**0.004 ± 0.004**	0.020 ± 0.027
**C. ETH_ID**					
Accuracy	0.992 ± 0.010	**0.998 ± 0.003**	0.994 ± 0.008	0.997 ± 0.002	0.997 ± 0.006
F1-score	0.343 ± 0.211	**0.538 ± 0.232**	0.327 ± 0.165	0.430 ± 0.167	0.494 ± 0.214
Precision	0.239 ± 0.186	**0.420 ± 0.245**	0.212 ± 0.146	0.293 ± 0.147	0.385 ± 0.236
Recall	0.956 ± 0.093	0.956 ± 0.057	**0.985 ± 0.038**	0.982 ± 0.054	0.938 ± 0.112
ROC AUC	0.999 ± 0.001	0.997 ± 0.009	**1.000 ± 0.000**	0.998 ± 0.004	0.999 ± 0.003
PR AUC	0.791 ± 0.126	0.786 ± 0.127	0.805 ± 0.082	0.744 ± 0.120	**0.839 ± 0.117**
MCC	0.433 ± 0.179	**0.600 ± 0.183**	0.431 ± 0.139	0.517 ± 0.130	0.562 ± 0.172
BER	0.026 ± 0.045	0.023 ± 0.028	0.010 ± 0.018	**0.010 ± 0.027**	0.033 ± 0.055
**D. MATRICE**					
Accuracy	0.974 ± 0.021	0.979 ± 0.016	0.976 ± 0.018	0.979 ± 0.013	**0.981 ± 0.010**
F1-score	0.162 ± 0.043	0.192 ± 0.044	0.180 ± 0.050	0.170 ± 0.055	**0.227 ± 0.050**
Precision	0.110 ± 0.039	0.136 ± 0.047	0.123 ± 0.047	0.117 ± 0.047	**0.156 ± 0.052**
Recall	0.407 ± 0.130	0.431 ± 0.139	0.456 ± 0.149	0.407 ± 0.161	**0.501 ± 0.116**
ROC AUC	0.834 ± 0.040	0.829 ± 0.039	0.804 ± 0.067	0.791 ± 0.088	**0.861 ± 0.028**
PR AUC	0.100 ± 0.018	0.116 ± 0.023	0.119 ± 0.031	0.123 ± 0.092	**0.155 ± 0.031**
MCC	0.192 ± 0.027	0.223 ± 0.031	0.217 ± 0.037	0.201 ± 0.055	**0.264 ± 0.033**
BER	0.308 ± 0.056	0.294 ± 0.063	0.283 ± 0.067	0.305 ± 0.075	**0.258 ± 0.053**

Continued on next page

Table S1 – continued from previous page

Metric	BERT-base	DeBERTa-v3	RoBERTa-base	RoBERTa-large	SciBERT
**E. SAMPLES**					
Accuracy	0.979 ± 0.015	**0.983 ± 0.005**	0.979 ± 0.012	0.979 ± 0.007	0.978 ± 0.021
F1-score	0.198 ± 0.033	0.253 ± 0.030	0.224 ± 0.038	0.226 ± 0.025	**0.265 ± 0.054**
Precision	0.182 ± 0.072	0.221 ± 0.057	0.194 ± 0.068	0.176 ± 0.041	**0.235 ± 0.087**
Recall	0.283 ± 0.102	0.335 ± 0.078	0.346 ± 0.122	0.356 ± 0.081	**0.392 ± 0.118**
ROC AUC	0.759 ± 0.051	0.775 ± 0.027	0.760 ± 0.053	0.743 ± 0.057	**0.814 ± 0.038**
PR AUC	0.113 ± 0.017	0.148 ± 0.024	0.145 ± 0.028	0.129 ± 0.022	**0.190 ± 0.032**
MCC	0.204 ± 0.021	0.257 ± 0.024	0.236 ± 0.027	0.236 ± 0.021	**0.277 ± 0.036**
BER	0.366 ± 0.043	0.338 ± 0.036	0.335 ± 0.055	0.330 ± 0.037	**0.313 ± 0.051**
**F. NUM_SAMPLES**					
Accuracy	0.988 ± 0.010	0.989 ± 0.006	0.985 ± 0.011	0.938 ± 0.220	**0.991 ± 0.004**
F1-score	0.251 ± 0.073	0.307 ± 0.061	0.250 ± 0.065	0.281 ± 0.107	**0.328 ± 0.048**
Precision	0.221 ± 0.117	0.216 ± 0.065	0.163 ± 0.055	0.198 ± 0.101	**0.244 ± 0.082**
Recall	0.449 ± 0.168	0.612 ± 0.115	0.630 ± 0.098	**0.654 ± 0.131**	0.617 ± 0.147
ROC AUC	0.872 ± 0.071	0.931 ± 0.038	0.912 ± 0.047	0.894 ± 0.103	**0.948 ± 0.039**
PR AUC	0.190 ± 0.051	0.260 ± 0.043	0.277 ± 0.065	0.248 ± 0.073	**0.334 ± 0.055**
MCC	0.282 ± 0.056	0.350 ± 0.038	0.307 ± 0.044	0.328 ± 0.095	**0.371 ± 0.032**
BER	0.280 ± 0.080	0.199 ± 0.055	**0.192 ± 0.044**	0.203 ± 0.085	0.196 ± 0.072
