# Supplementary material for: The silence of the labs: Ethical oversight in metabolomics research
Source: PLoS One. 2026 Oct 7;21(10):e0346284. doi: 10.1371/journal.pone.0346284 (PMC13645098; doi:10.1371/journal.pone.0346284)
Supplement: Supplementary file 2 [file pone.0346284.s009.pdf]

Table S2: Omnibus comparisons of transformer architectures based on positive-class F1-score. Friedman tests were performed across five architectures using matched results from 20 random-seed runs. Kendall's W is reported as the effect-size measure.

**Analysis**	χ^2	**df**	p**-value**	**Kendall's** W
Overall ethics macro-F1	22.60	4	< 0.001	0.283
Experimental-context macro-F1	36.08	4	< 0.001	0.451
ETHICAL	15.40	4	0.0039	0.193
ETH_BOARD	9.89	4	0.0423	0.124
ETH_ID	10.52	4	0.0325	0.131
MATRICE	22.32	4	< 0.001	0.279
SAMPLES	36.80	4	< 0.001	0.460
NUM_SAMPLES	16.72	4	0.0022	0.209
