# Supplementary material for: The silence of the labs: Ethical oversight in metabolomics research
Source: PLoS One. 2026 Oct 7;21(10):e0346284. doi: 10.1371/journal.pone.0346284 (PMC13645098; doi:10.1371/journal.pone.0346284)
Supplement: Supplementary file 3 [file pone.0346284.s010.pdf]

Table S3: Holm-adjusted pairwise Wilcoxon signed-rank comparisons of positive-class F1-score among transformer architectures. Comparisons used matched results from 20 random-seed runs. Holm correction was applied separately within each analysis across the 10 model-pair comparisons. Raw and Holm-adjusted p-values, mean paired differences, and matched-pairs rank-biserial correlations are reported. Mean difference is defined as Model A minus Model B; positive values therefore favor Model A and negative values favor Model B. Bold adjusted p-values indicate statistical significance at $\alpha = 0.05$.

**Model A**	**Model B**	W	**Raw** p	**Holm** p	**Mean diff.**	r_{rb}
**Overall ethics macro-F1**						
BERT-base	DeBERTa-v3	80.0	0.3683	1.0000	+0.050	+0.238
BERT-base	RoBERTa-base	70.0	0.2024	1.0000	+0.047	+0.333
BERT-base	RoBERTa-large	104.0	0.9854	1.0000	0.000	-0.010
BERT-base	SciBERT	28.0	0.0027	**0.0217**	-0.111	-0.733
DeBERTa-v3	RoBERTa-base	102.0	0.9273	1.0000	-0.003	+0.029
DeBERTa-v3	RoBERTa-large	77.0	0.3118	1.0000	-0.050	-0.267
DeBERTa-v3	SciBERT	18.0	< 0.001	**0.0043**	-0.160	-0.829
RoBERTa-base	RoBERTa-large	75.0	0.2774	1.0000	-0.047	-0.286
RoBERTa-base	SciBERT	11.0	< 0.001	**0.0010**	-0.158	-0.895
RoBERTa-large	SciBERT	28.0	0.0027	**0.0217**	-0.110	-0.733
**Experimental-context macro-F1**						
BERT-base	DeBERTa-v3	8.0	< 0.001	< 0.001	-0.047	-0.924
BERT-base	RoBERTa-base	61.0	0.1054	0.2108	-0.015	-0.419
BERT-base	RoBERTa-large	41.0	0.0153	0.0766	-0.022	-0.610
BERT-base	SciBERT	1.0	< 0.001	< 0.001	-0.070	-0.990
DeBERTa-v3	RoBERTa-base	26.0	0.0020	**0.0139**	+0.032	+0.752
DeBERTa-v3	RoBERTa-large	47.0	0.0296	0.1183	+0.025	+0.552
DeBERTa-v3	SciBERT	50.0	0.0400	0.1200	-0.022	-0.524
RoBERTa-base	RoBERTa-large	80.0	0.3683	0.3683	-0.007	-0.238
RoBERTa-base	SciBERT	15.0	< 0.001	**0.0021**	-0.055	-0.857
RoBERTa-large	SciBERT	26.0	0.0020	**0.0139**	-0.048	-0.752
**ETHICAL**						
BERT-base	DeBERTa-v3	45.0	0.0240	0.1677	+0.125	+0.571
BERT-base	RoBERTa-base	60.0	0.0973	0.4953	+0.125	+0.429
BERT-base	RoBERTa-large	77.0	0.3118	1.0000	+0.062	+0.267
BERT-base	SciBERT	58.0	0.0826	0.4953	-0.088	-0.448
DeBERTa-v3	RoBERTa-base	101.0	0.8983	1.0000	0.000	-0.038
DeBERTa-v3	RoBERTa-large	79.0	0.3488	1.0000	-0.062	-0.248
DeBERTa-v3	SciBERT	22.0	0.0010	**0.0102**	-0.213	-0.790
RoBERTa-base	RoBERTa-large	78.0	0.3300	1.0000	-0.063	-0.257
RoBERTa-base	SciBERT	26.0	0.0020	**0.0179**	-0.213	-0.752
RoBERTa-large	SciBERT	27.0	0.0023	**0.0186**	-0.150	-0.743

Continued on next page

Table S3 – continued from previous page

**Model A**	**Model B**	W	**Raw** p	**Holm** p	**Mean diff.**	r_{rb}
**ETH_BOARD**						
BERT-base	DeBERTa-v3	41.0	0.0153	0.1225	+0.220	+0.610
BERT-base	RoBERTa-base	95.0	0.7285	1.0000	0.000	-0.095
BERT-base	RoBERTa-large	90.0	0.8405	1.0000	+0.024	+0.053
BERT-base	SciBERT	62.0	0.1140	0.6839	-0.093	-0.410
DeBERTa-v3	RoBERTa-base	35.0	0.0073	0.0657	-0.220	-0.667
DeBERTa-v3	RoBERTa-large	44.0	0.0215	0.1504	-0.196	-0.581
DeBERTa-v3	SciBERT	21.0	< 0.001	**0.0085**	-0.312	-0.800
RoBERTa-base	RoBERTa-large	95.0	0.7285	1.0000	+0.024	+0.095
RoBERTa-base	SciBERT	66.0	0.2432	1.0000	-0.093	-0.305
RoBERTa-large	SciBERT	82.0	0.4091	1.0000	-0.117	-0.219
**ETH_ID**						
BERT-base	DeBERTa-v3	40.0	0.0136	0.1089	-0.195	-0.619
BERT-base	RoBERTa-base	102.0	0.9273	0.9273	+0.016	+0.029
BERT-base	RoBERTa-large	70.0	0.2024	0.7682	-0.087	-0.333
BERT-base	SciBERT	51.0	0.0441	0.3084	-0.151	-0.514
DeBERTa-v3	RoBERTa-base	26.0	0.0020	**0.0199**	+0.211	+0.752
DeBERTa-v3	RoBERTa-large	66.0	0.1536	0.7682	+0.108	+0.371
DeBERTa-v3	SciBERT	81.0	0.3884	0.7768	+0.044	+0.229
RoBERTa-base	RoBERTa-large	52.0	0.0484	0.3084	-0.103	-0.505
RoBERTa-base	SciBERT	32.0	0.0049	**0.0437**	-0.167	-0.695
RoBERTa-large	SciBERT	67.0	0.1650	0.7682	-0.064	-0.362
**MATRICE**						
BERT-base	DeBERTa-v3	48.0	0.0328	0.2294	-0.031	-0.543
BERT-base	RoBERTa-base	69.0	0.1893	0.7574	-0.018	-0.343
BERT-base	RoBERTa-large	83.0	0.4304	0.7833	-0.008	-0.210
BERT-base	SciBERT	15.0	< 0.001	**0.0026**	-0.065	-0.857
DeBERTa-v3	RoBERTa-base	79.0	0.3488	0.7833	+0.012	+0.248
DeBERTa-v3	RoBERTa-large	65.0	0.1429	0.7145	+0.022	+0.381
DeBERTa-v3	SciBERT	56.0	0.0696	0.4175	-0.035	-0.467
RoBERTa-base	RoBERTa-large	74.0	0.2611	0.7833	+0.010	+0.295
RoBERTa-base	SciBERT	39.0	0.0121	0.0966	-0.047	-0.629
RoBERTa-large	SciBERT	29.0	0.0032	**0.0284**	-0.057	-0.724

Continued on next page

Table S3 – continued from previous page

**Model A**	**Model B**	W	**Raw** p	**Holm** p	**Mean diff.**	r_{rb}
**SAMPLES**						
BERT-base	DeBERTa-v3	5.0	< 0.001	< 0.001	-0.055	-0.952
BERT-base	RoBERTa-base	47.0	0.0296	0.0887	-0.026	-0.552
BERT-base	RoBERTa-large	19.0	< 0.001	**0.0053**	-0.028	-0.819
BERT-base	SciBERT	19.0	< 0.001	**0.0053**	-0.067	-0.819
DeBERTa-v3	RoBERTa-base	33.0	0.0056	**0.0279**	+0.029	+0.686
DeBERTa-v3	RoBERTa-large	27.0	0.0023	**0.0163**	+0.027	+0.743
DeBERTa-v3	SciBERT	66.0	0.1536	0.3073	-0.011	-0.371
RoBERTa-base	RoBERTa-large	105.0	1.0000	1.0000	-0.002	+0.000
RoBERTa-base	SciBERT	41.0	0.0153	0.0612	-0.040	-0.610
RoBERTa-large	SciBERT	31.0	0.0042	**0.0253**	-0.039	-0.705
**NUM_SAMPLES**						
BERT-base	DeBERTa-v3	50.0	0.0400	0.2799	-0.056	-0.524
BERT-base	RoBERTa-base	104.0	0.9854	1.0000	0.000	+0.010
BERT-base	RoBERTa-large	70.0	0.2024	1.0000	-0.030	-0.333
BERT-base	SciBERT	21.0	< 0.001	**0.0077**	-0.077	-0.800
DeBERTa-v3	RoBERTa-base	34.0	0.0064	0.0511	+0.057	+0.676
DeBERTa-v3	RoBERTa-large	82.0	0.4091	1.0000	+0.026	+0.219
DeBERTa-v3	SciBERT	81.0	0.3884	1.0000	-0.021	-0.229
RoBERTa-base	RoBERTa-large	79.0	0.3488	1.0000	-0.030	-0.248
RoBERTa-base	SciBERT	18.0	< 0.001	**0.0048**	-0.077	-0.829
RoBERTa-large	SciBERT	68.0	0.1769	1.0000	-0.047	-0.352
