# Supplementary material for: The silence of the labs: Ethical oversight in metabolomics research
Source: PLoS One. 2026 Oct 7;21(10):e0346284. doi: 10.1371/journal.pone.0346284 (PMC13645098; doi:10.1371/journal.pone.0346284)
Supplement: Supplementary file 4 [file pone.0346284.s013.pdf]

Answers to Reviewers

The Silence of the Labs: Ethical Oversight in Metabolomics Research

Joan Badia[1†], Maria Llambrich[1,2†], Rocío López-Rubio[2], Horacio Saggion[3*], Raquel Cumeras[1,2*]

Dear Editor and Reviewers,

We sincerely thank you for your careful evaluation of our manuscript and for the constructive comments. We have addressed each point in detail and believe that the revisions have substantially improved the clarity, methodological transparency, statistical robustness, and scope of the manuscript.

In addition to the substantive revisions described below, the manuscript has been **reformatted** to comply with the PLOS ONE template and journal style requirements. These formatting changes include adjustments to the manuscript structure, section headings, presentation of tables and figures, and supporting-information organization. As these modifications are editorial rather than substantive, they are not discussed individually in the point-by-point responses. Purely formatting-related modifications have not been highlighted as tracked changes unless they coincided with substantive revisions to the text.

Reviewer comments are reproduced below, followed by our point-by-point responses.

Reviewer #1:

Authors have described a promising work on "The Silence of the Labs: Ethical Oversight in Metabolomics Research" The current manuscript sounds good, however, I have noted the major concerns listed below. Authors should address all raised points/comments before considering the manuscript to this journal.

Thank you for reviewing our manuscript. We believe that the revisions made have substantially improved the manuscript and have adequately addressed all the comments and concerns raised by the reviewers. We hope that the revised version is now suitable for publication in PLOS ONE.

Major Comments

1. The manuscript lacks sufficient context on existing tools or frameworks for detecting ethical statements in published literature. A comparative approach

would help readers understand how MinEth differs from previously reported works. The authors are suggested to expand the introduction or methodology section where tools, frameworks and techniques in the field of ethics detection in research articles would be discussed. This would help in strengthening the value of this article in terms of novelty.

We agree with the reviewer and we have expanded the Introduction to provide a broader and more comparative context on existing biomedical text-mining tools, reporting-quality frameworks, and ethics-related NLP resources. Specifically, the revised manuscript now discusses prior biomedical meta-research on ethics approval and informed consent reporting, including studies that manually assessed the presence or absence of ethics statements across different biomedical fields. We also added a new comparative table summarizing these studies to contextualize the reporting gap addressed by MinEth.

In addition, we expanded the Introduction to describe related NLP resources and tools for extracting structured information from biomedical publications, including PICO extraction corpora and systems, Trialstreamer, RobotReviewer, method-entity extraction approaches, and transformer-based models used for biomedical and scientific text mining. We also included recent ethics-related NLP work, such as resources focused on ethical concern statements in AI/NLP literature.

These additions clarify that previous tools and frameworks mainly address clinical evidence extraction, trial characteristics, methodological reporting, metadata extraction, reporting quality, risk-of-bias assessment, or ethical concerns in AI/NLP papers. In contrast, MinEth specifically focuses on sentence-level detection of ethical approval statements, ethics committee or institutional review board names, and formal approval identifiers in full-text human metabolomics articles. This comparison strengthens the rationale and novelty of MinEth as, to our knowledge, the first benchmark dedicated to automated detection of ethical oversight reporting in metabolomics literature.

2. The methodology section of this manuscript lacks detail on the annotation workflow. The reliability of annotations is mentioned but not explained in detail. Providing a more detailed explanation of the data labelling process like number of annotators, criteria used for identifying ethical statement, challenges and how to resolve them, etc. would help readers get the best out of this article.

We agree with the reviewer that the annotation workflow needed to be improved. We have expanded the Methods section to provide a more detailed description of the annotation workflow and data-labelling process.

NEW TEXT: "To minimize interpretation bias and ensure consistency, annotations were conducted independently and blindly on overlapping documents, with no access to other annotators' labels during the initial round. Ethical statements were identified at the sentence level using predefined operational criteria: sentences were labeled ETHICAL if they explicitly referred to ethics approval, institutional review, informed consent, or approval waiver; ETH_BOARD if they mentioned an ethics committee or institutional review board; and ETH_ID if they included an approval number or protocol identifier. Annotators, with complementary expertise in biomedical text mining, metabolomics, and ethics curation, were trained using a shared guideline and a pilot set of articles to harmonize label interpretation, particularly for ambiguous cases.

The annotation task was challenging because ethics-related information was sparse and heterogeneously reported across full-text articles. Relevant statements were not restricted to a single section and could appear in Methods, Acknowledgments, ethics declarations, data-availability sections, or Supplementary Materials. In addition, several sentences contained multiple ethics-related elements, whereas others provided only partial information, such as a committee name without an approval identifier or a consent statement without explicit ethics approval. Disagreements mainly involved boundary decisions between general ethics approval mentions, named oversight bodies, and formal identifiers. These cases were reviewed during adjudication, and a senior researcher harmonized tag usage, corrected boundary inconsistencies, and produced the final consolidated annotations used for model training and evaluation."

3. The performance metrics for the transformer models should be clearly visualized. The authors would be suggested to add a table that would summarize the performance of BERT, RoBERTa, SciBERT, DeBERTa-v3 across various ethical categories, allowing for easier comparison of results and would make findings more accessible to readers.

We thank the reviewer for this useful suggestion. We added a category-level summary table reporting mean ± standard deviation across 20 independent fine-tuning runs. The revised Table 5 shows that SciBERT achieved the highest mean F1 for ETHICAL (0.707 ± 0.112) and ETH_BOARD (0.640 ± 0.195), as well as the highest overall ethics macro-F1 (0.614 ± 0.111). DeBERTa-v3 achieved the highest mean F1 for ETH_ID (0.538 ± 0.232), although it did not significantly outperform all competing architectures after Holm correction. The table caption now clarifies that bold values indicate the highest mean and do not necessarily represent statistically significant pairwise differences.

4. The article lacks a comparative study of transformer models with traditional manual methods of detecting ethics-related statements. Providing a brief comparison of the performance of transformer models with traditional manual annotation techniques in terms of accuracy, speed and scalability would strengthen the claims about the utility of the automated system.

In the original version, the distinction between manual annotation and automated transformer-based classification may not have been sufficiently explicit. We have revised the manuscript to clarify that MinEth follows a two-stage design: first, expert manual annotation in GATE was used to create an adjudicated gold-standard corpus; second, transformer-based models were trained as supervised sentence classifiers using this manually curated corpus. Therefore, the transformer models were not used to generate the reference annotations, but to evaluate whether manually defined ethics-related categories could be detected automatically and reproducibly at scale.

At the end of the introduction, we have changed a sentence:

FROM: "Sentences in the corpus are labeled with ethics-related categories (e.g., ETHICAL approval mention, ETH ID identifiers, ETH BOARD names, consent statements). Using this corpus, we fine-tune and compare models including BERT, RoBERTa, SciBERT, and DeBERTa to detect ethical statements and their subtypes automatically."

TO: "Sentences in the corpus are labeled with ethics-related categories (e.g., ETHICAL approval mention, ETH_ID identifiers, ETH_BOARD names, consent statements), and experimental context (MATRICE, SAMPLES, NUM_SAMPLES). Using this manually curated corpus, we trained and evaluated supervised transformer-based sentence classifiers including BERT, RoBERTa, SciBERT, and DeBERTa to detect statements and their subtypes automatically, repeating each model–task experiment across 20 random seeds to quantify stochastic training variability."

And added the following text to the DISCUSSION: "Manual annotation and automated detection played complementary roles in this study. Manual annotation in GATE provided the expert-derived reference standard used to train and evaluate the supervised transformer classifiers. This process offered high interpretability and reliability, as reflected by the inter-annotator agreement, but required expert review of full-text articles and remained difficult to scale. This limitation was especially relevant because ethics-related statements represented less than 1% of all sentences and appeared in heterogeneous locations across articles, including Methods, Acknowledgments, ethics declarations, and Supplementary Materials. In contrast, once trained on the adjudicated corpus, transformer-based sentence classifiers enabled rapid and reproducible screening of large sentence collections.

Thus, MinEth does not replace manual expert assessment; rather, it converts manual annotation into a reusable benchmark that supports scalable detection of ethics-related reporting across the literature."

Minor Comments

1. The article consists of technical terms and concepts that are not sufficiently explained for readers who may not be familiar with NLP or machine learning methods.

We have revised the manuscript to improve accessibility for readers who may not be familiar with NLP or machine-learning terminology. Specifically, we added brief explanations of supervised learning, sentence-level classification, precision, recall/sensitivity, F1-score, ROC AUC, PR AUC, balanced error rate, and macro-averaged metrics.

2. A more detailed explanation should be included regarding how **Cohen's Kappa** was calculated and any potential limitations or uncertainties in the annotation process should be discussed. **Annotation bias should also be reported**.

We have expanded the methods section *5.4 Inter-Annotator Agreement* to clarify how Cohen's κ was calculated. Additionally, we have included how Fleiss κ was calculated.

We also expanded the Discussion to address annotation uncertainty, potential curator-dependent bias, and the limitations of the partially overlapping annotation design.

BEFORE: "Although annotation consistency was high Cohen's κ = 0.84, differences in sentence interpretation could influence boundary decisions between approval mentions and identifiers."

NOW: "Although annotation consistency was high Cohen's κ = 0.84, some annotation uncertainty and potential annotation bias remain. Relevant ethics-related information was sparse and often distributed across different sections of the article, which could lead annotators to select different but semantically related spans. In addition, although annotators were trained using shared guidelines, this common training and domain expertise may have introduced interpretation bias toward similar readings of ambiguous statements. The use of independent blind annotation, predefined operational criteria, and subsequent adjudication was intended to minimize these effects, but cannot completely eliminate curator-dependent decisions."

3. The article is well organized but could benefit from clearer **subsection headings** within the Methodology section.

We thank the reviewer for this helpful suggestion. We have revised the subsection headings within the Materials and Methods section to make them more descriptive and to better reflect the content of each methodological step.

4. Although the abstract highlights the importance of MinEth for scalable and reproducible monitoring of ethical reported, but **the potential broader applications have not been fully discussed in the main text**.

We thank the reviewer for this important observation. We agree that the broader applications of MinEth were not sufficiently developed in the main text. We have expanded the Discussion to clarify how MinEth could be applied beyond the present benchmark, including journal editorial screening, funder and institutional compliance monitoring, research-integrity audits, repository metadata enrichment, and extension to other omics or biomedical domains. We also clarified that such systems should be considered decision-support tools and not replacements for human ethical review.

BEFORE: "From a broader perspective, MinEth illustrates how natural language processing can complement human oversight in research governance. Automated screening tools derived from this framework could assist editors, reviewers, and institutions in verifying compliance with ethical requirements at scale. Integrating such tools into submission systems or data repositories would enhance accountability and facilitate machine-readable ethics metadata across scientific domains. As large language models become increasingly embedded in academic workflows, transparent datasets like MinEth are essential for ensuring that automation reinforces, rather than replaces, ethical responsibility in research reporting.

NOW: "Beyond metabolomics, MinEth could support broader biomedical research governance by helping editors, reviewers, funders, and institutions identify articles in which ethics-related information is missing, incomplete, or difficult to locate. This includes the absence of reported ethics committee or institutional review board approval, consent statements, and approval identifiers. Similar models could also enrich repository metadata by making ethical oversight information more searchable and machine-readable. These applications should be viewed as decision-support tools for scalable monitoring, not as replacements for human ethical review."

Reviewer #2:

The paper explores the **automated detection** and categorization of ethics-related information from metabolomics publications. The paper proposes the MinEth dataset for ethics reporting analysis, based on which to develop **automated sentence-level classifiers**. The research question is **timely** and the MinEth benchmark corpus is a **great contribution**, with open data and code availability that aligns well with PLOS ONE's open science values. However, the manuscript has problems with writing quality, statistical analysis, annotation process clarity, strength of results, and coverage of discussion.

Thank you for reviewing our manuscript. We believe that the revisions made have substantially improved the manuscript and have adequately addressed all the comments and concerns raised by the reviewers. We hope that the revised version is now suitable for publication in PLOS ONE.

The detailed comments:

a. Inconsistent logic in result paragraphs. Problems exist in the following paragraph: "The detailed metrics of DeBERTa-v3 (Figure 2) illustrate good F1 performance across ETH ID (0.681) categories. However, lower F1scores (< 0.35) for ETH ID and control categories of experiment context (MATRICE, SAMPLES, and NUM SAMPLES). These inconsistencies suggest that while SciBERT effectively captures linguistic patterns associated with ethics oversight, its ability to generalize to numerically dominated or structurally irregular tokens remains limited." Firstly, it is not clear why the paragraph opens by discussing DeBERTa but closes with a conclusion naming SciBERT. Additionally, the sentence "lower F1scores (< 0.35) for ETH ID and control categories of experiment context (MATRICE, SAMPLES, and NUM SAMPLES)" is incomplete. Furthermore, the first sentence mentions "DeBERTa-v3 illustrates good F1 performance across ETH ID", while the second sentence mentions "lower F1scores (< 0.35) for ETH ID", which directly **contradicts the first sentence**.

We thank the reviewer for identifying this inconsistency. The original paragraph was removed and the Results section was restructured following the repeated-run analysis. The revised manuscript no longer interprets the performance of DeBERTa-v3 from a single run. Instead, Figure 1 and Tables 4–6 report mean performance and standard deviation across 20 random seeds.

The revised results show that SciBERT achieved the strongest overall ethics macro-F1 and the highest mean F1 for ETHICAL and ETH_BOARD, whereas DeBERTa-v3 achieved the highest mean F1 for ETH_ID without significantly outperforming all competing architectures. Experimental-context performance is now discussed

separately and is explicitly described as modest. This restructuring removes the contradictory statements and ensures that the interpretation consistently reflects the repeated-run statistical analysis.

b. Figure reference errors. The paper contains several error references as "Figure ?? S1", "Figure ?? S2", "Figure ?? S3", and "Figure ?? S4" in the Results section.

We thank the reviewer for pointing out these figure-reference errors. We have corrected them.

c. Inconsistent content between introduction and results. The Introduction states the paper will "**analyze trends over journals, publication years, and compliance levels**." However, no such analysis is presented in the Results or Discussion sections.

We thank the reviewer for pointing out this inconsistency. To address this, we have added a new article-level analysis at the end of the Results section. For each article, we assessed the presence of ETHICAL, ETH_BOARD, and ETH_ID annotations and summarized ethics-reporting completeness across journals and publication years.

We added a new Results subsection entitled "Article-level ethics-reporting completeness across journals and years". With the text:

"Finally, we assessed article-level patterns in ethics reporting across the 120 articles in the MinEth corpus. Overall, 83 articles (69.2%) reported ethics-related information, 70 (58.3%) reported an ethics committee or institutional review board, and 37 (30.8%) reported a formal approval identifier. Complete ethics reporting, defined as the presence of all three elements, was observed in 37 articles (30.8%), whereas 37 articles (30.8%) contained no reported ethics-related information.

Ethics-reporting completeness varied across journals, indicating heterogeneity in reporting practices (Figure 2). Temporal analysis suggested that approval identifiers and complete reporting were more frequent in recent years, particularly from 2018 onwards. When grouped into publication periods, general ethics-related reporting remained relatively stable, with ETHICAL annotations present in 66.7% of articles published in 2007–2013, 67.6% in 2014–2017, and 70.4% in 2018–2021. Ethics committee or institutional review board reporting also remained similar across periods (60.0%, 55.9%, and 59.2%, respectively). In contrast, formal approval identifiers were more frequently reported in the most recent period, increasing from 26.7% in 2007–2013 and 14.7% in 2014–2017 to 39.4% in 2018–2021. Complete ethics reporting followed the same pattern, reaching 39.4% in 2018–2021 compared

with 26.7% and 14.7% in the earlier periods. Articles with no reported ethics-related information persisted across all periods at ~30%.

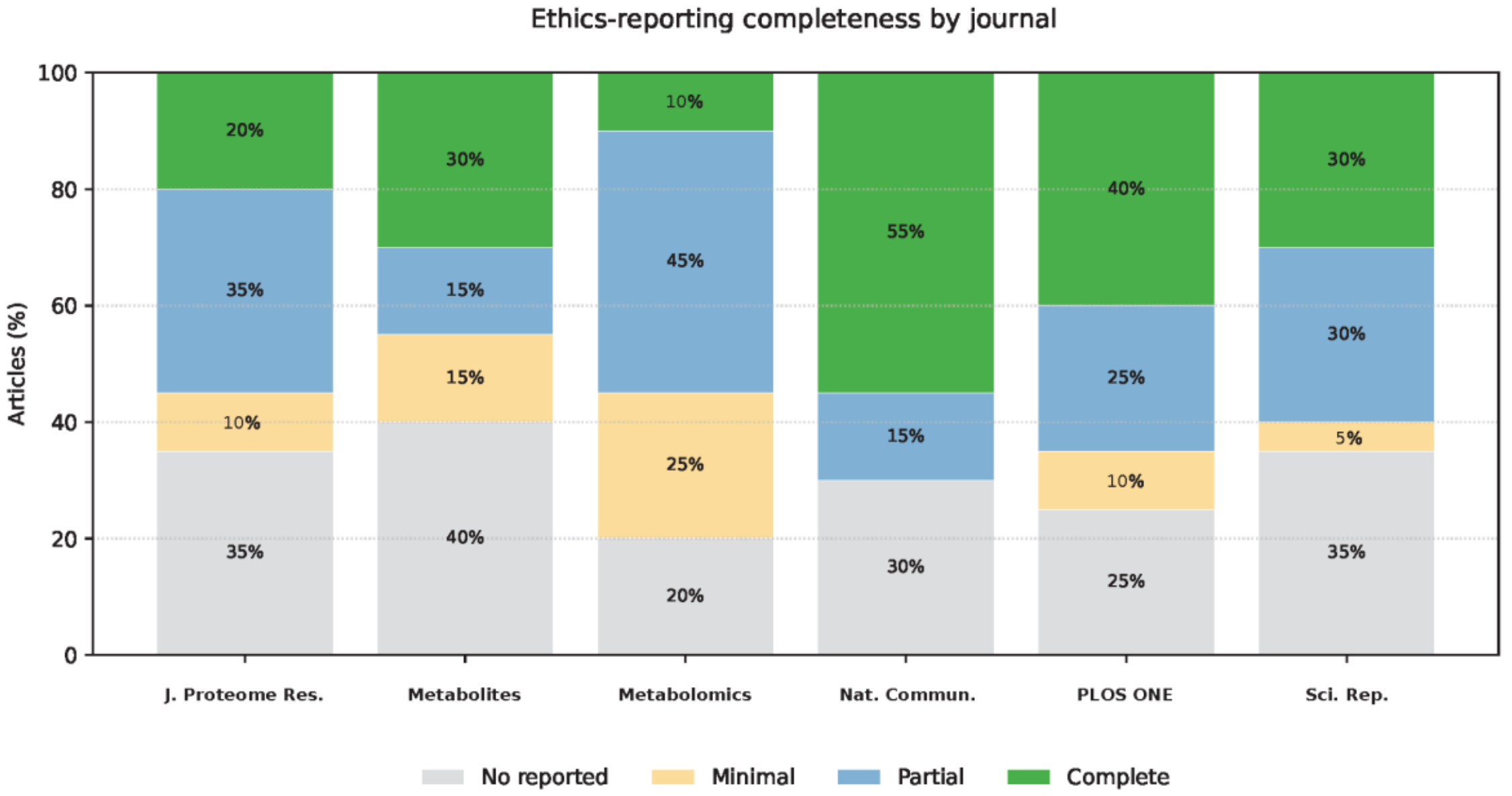

Figure 2: Ethics-reporting completeness by journal in the MinEth corpus. Articles were classified according to the presence of ETHICAL, ETH_BOARD, and ETH_ID annotations. Complete reporting indicates the presence of all three elements; partial reporting indicates an ethics-related statement together with either an ethics board mention or an approval identifier; minimal reporting indicates an ethics statement only; and no reported ethics information indicates absence of all three elements."

d. Unclear annotation process. The annotation methodology has several gaps:

1. Annotator **qualifications** are not clear in Sections 5.3 and 5.4. Further detailed information regarding annotator roles (e.g., graduate student, or domain expert), level of expertise, and training guidelines would be helpful.

We thank the reviewer for this helpful comment. We have revised Sections 5.3 and 5.4 to provide clearer information about the annotator roles, expertise, and training procedure. The revised manuscript now specifies that annotation was performed by trained annotators with complementary expertise in biomedical text mining, metabolomics, and ethics-reporting curation. We also clarified that annotators followed predefined written guidelines and completed a pilot training phase before the main annotation round.

NEW: "To minimize interpretation bias and ensure consistency, annotations were conducted independently and blindly on overlapping documents, with no access to other annotators' labels during the initial round. Ethical statements were identified

at the sentence level using predefined operational criteria: sentences were labeled ETHICAL if they explicitly referred to ethics approval, institutional review, informed consent, or approval waiver; ETH_BOARD if they mentioned an ethics committee or institutional review board; and ETH_ID if they included an approval number or protocol identifier. Annotators, with complementary expertise in biomedical text mining, metabolomics, and ethics curation, were trained using a shared guideline and a pilot set of articles to harmonize label interpretation, particularly for ambiguous cases."

2. Section 5.4 mentions **Fleiss' κ** is calculated, while this statistic is not reported anywhere in the paper.

Thank you for pointing this out. We have now reported Fleiss' κ in the Results section *2.2 Annotation Reliability*.

NEW TEXT:

"In the triple-annotated subset, agreement was perfect for the ETHICAL category alone (Fleiss' $\kappa = 1.00$), indicating that explicit ethics statements were consistently identified. Lower agreement for secondary and experimental-context categories (Fleiss' κ = 0.42-0.60) reflected the fact that relevant information was often distributed across different sections of the articles, including abstracts, methods, sample collection descriptions, and ethics statements. As a result, annotators sometimes selected different but semantically related spans, or differed in whether board names, identifiers, sample counts, or matrices were annotated as independent entities. The final corpus was reviewed and harmonized before model training to correct boundary mismatches and ensure consistent use of annotation labels."

3. It is still not clear how the corpus is divided among three annotators. Section 5.3 mentions "annotated 56 articles each, with 32 overlapping documents to measure inter-annotator consistency. Twenty-four articles (20%) were triple-annotated to enable adjudication and reliability assessment." The use of different overlap levels (double vs. triple annotation) for different subsets means the **comparison base for inter-annotator agreement is not uniform**.

We agree that the annotation coverage is not fully uniform across the corpus. The design intentionally used a double-annotated subset for pairwise consistency checks and a smaller triple-annotated subset for adjudication and reliability assessment, so agreement statistics were computed on different overlap structures. This was done because triple annotation is more resource-intensive, but

we recognize that it makes the comparison base heterogeneous. We have added a clarification sentence regarding the Cohen's κ and Fleiss' κ.

NEW: "Because the corpus was annotated using a partially overlapping design, Cohen's κ and Fleiss' κ were calculated on different annotation subsets and were interpreted as complementary reliability estimates rather than as directly interchangeable measures."

4. It is not mentioned how disagreements among annotators are resolved or how annotator agreement is improved.

We have expanded the annotation workflow to clarify how disagreements were handled. The revised manuscript now explains that disagreements mainly involved boundary decisions between general ethics approval mentions, named oversight bodies, and formal identifiers. These cases were reviewed during adjudication, and a senior researcher harmonized tag usage, corrected boundary inconsistencies, and produced the final consolidated annotations used for model training and evaluation. We also clarified that annotators were first trained using a pilot set of documents to harmonize interpretation of the labeling criteria before the main annotation round.

5. It is not clear how to come up with the categories (e.g., approval mentions and institutional review boards). Are the current categories enough to cover all cases?

We have clarified in Section 5.3 that the annotation categories were defined a priori from the reporting elements needed to assess traceable ethical oversight in human metabolomics publications. We agree that the current categories are not intended to cover all possible ethics-governance information, and we have clarified this in the discussion section.

NEW text in Section 5.3: "Annotation categories were defined a priori to capture the minimum information required to assess traceable ethical oversight in human metabolomics studies. Ethics-related categories included: ETHICAL, for explicit statements of ethics approval, informed consent, exemption, or waiver (e.g., "The study was approved by the Institutional Review Board"); ETH_BOARD, for the name of the approving ethics committee or institutional review board (e.g., "Somerset Research and Ethics Committee"); and ETH_ID, for formal approval numbers, protocol codes, or equivalent identifiers (e.g., "LREC Protocol Number: 05/Q2202/96"). Together, these categories determine whether ethical oversight was reported, who provided it, and whether it is traceable. Additional experimental-context categories captured key study descriptors, including MATRICE (e.g.,

“plasma samples”), SAMPLES (e.g., “blood samples were collected”), and NUM_SAMPLES (e.g., “n = 26”).”

NEW text in Discussion: “Moreover, the current annotation schema was designed to capture the core elements required for traceable ethics reporting, namely whether ethical oversight was reported, which committee or institutional review board provided oversight, and whether a formal approval identifier was available. However, these categories are not intended to exhaustively cover all possible ethics-governance information. For example, detailed consent scope, data-sharing permissions, privacy safeguards, participant withdrawal rights, and secondary-use restrictions were not annotated as separate categories in the present version of MinEth.”

e. Lack of statistical significance analysis. All model comparisons are based on a single run. The model performance-related claims such as "DeBERTa-v3 substantially outperformed all other models" should be justified by statistical analysis.

We thank the reviewer for this important comment. We agree that conclusions based on a single fine-tuning run would not adequately account for the stochastic variability associated with transformer training. We therefore repeated every model–task experiment across 20 independent random seeds while maintaining identical preprocessing procedures, hyperparameters, and held-out test partitions.

Statistical comparisons were based on the positive-class F1-scores obtained across the 20 repeated runs. Because the same seeds and data partitions were used across architectures, seeds were treated as paired blocks. Differences among the five architectures were first assessed using the non-parametric Friedman test. When the omnibus test was significant, two-sided paired Wilcoxon signed-rank tests were performed for all model pairs, followed by Holm correction for multiple comparisons. Kendall’s W and matched-pairs rank-biserial correlations were also reported as effect-size measures.

The repeated-run analysis substantially changed the interpretation of the results. SciBERT achieved the highest overall ethics macro-F1 (0.614 ± 0.111) and significantly exceeded all other evaluated architectures after Holm correction. DeBERTa-v3 achieved the highest mean F1 for ETH_ID (0.538 ± 0.232), but it did not significantly outperform all competing models. We therefore removed the previous claim that DeBERTa-v3 “substantially outperformed all other models” and now distinguish between the highest observed mean and statistically supported pairwise superiority.

We have updated the Abstract, Methods, Results, Discussion, Conclusions, Tables 4–6, and Figure 1 accordingly. Complete repeated-run metrics, omnibus Friedman-test results, and Holm-adjusted pairwise comparisons are provided in Supporting Tables S1–S3. Per-seed results and the scripts used for aggregation and statistical testing have been deposited and are available in the project repository and data archive.

We have additionally clarified that the repeated-run comparisons quantify variability arising from stochastic fine-tuning under a fixed corpus partition, but do not capture uncertainty associated with alternative corpus samples or train/test splits. Future work should therefore include repeated cross-validation and external validation.

f. No analysis with large-Scale generative models. Inspired by the state-of-the-art performance of generative models (e.g., GPT family), it would be interesting to see the performance of generative models **on identifying ethics-related information and categorize it**.

We thank the reviewer for this valuable suggestion. We agree that evaluating instruction-tuned generative language models represents an important direction for future research. However, the present study was designed as a controlled supervised sentence-classification benchmark in which all architectures were trained using the same manually annotated corpus, fixed target categories, preprocessing procedures, and held-out evaluation partition.

To compare manual annotations with automated annotations, we chose to use language models to perform supervised sentence classification tasks, and not generative language models such as GPT family. Those models also would not fit on the NVIDIA RTX4080 we used for these analyses and we relied on smaller models that would fit on the VRAM. Limited fundings also restricted the use of cloud computing solutions to overcome our local computing limitations.

A rigorous comparison with generative models would require a separate experimental protocol addressing model and version selection, zero-shot versus few-shot prompting, prompt variability, decoding parameters, output normalization, computational cost, data privacy, and reproducibility over time. We therefore considered such an evaluation beyond the scope of the present revision rather than adding a limited comparison that would not be methodologically equivalent to the supervised experiments.

We have acknowledged this limitation and added generative-model evaluation as a future research direction.

g. Limited discussion (Section 3). The poor performance on experimental categories is not acknowledged in discussion. The best macro-F1 across all models for experimental classification is only 0.290. A complete discussion should address the practical utility of the framework considering the poor performance on experimental categories.

We agree that the practical limitations of the experimental-context classifiers required clearer discussion. Following the repeated-run analysis, the highest mean experimental-context macro-F1 was 0.273 ± 0.034 for SciBERT, rather than the single-run value of 0.290 reported in the original manuscript. Although SciBERT achieved the highest average performance, its macro-F1 was not significantly different from that of DeBERTa-v3 after Holm correction (p_{adj} = 0.120).

We have revised both the Results section and the Discussion and have updated Table 6 accordingly. The revised manuscript now explicitly states that performance for MATRICE, SAMPLES, and NUM_SAMPLES remained modest and variable across runs. These categories were included as auxiliary descriptors to provide experimental context and are not part of the three core categories used to assess traceable ethics reporting. Therefore, their lower performance does not alter the primary contribution of MinEth, but it limits the current utility of the framework for standalone extraction of sample matrices, sample descriptions, or sample-size information.

We now clarify that MinEth should primarily be considered a framework for ethics-reporting detection, whereas experimental-context extraction remains preliminary and requires further methodological development. The lower performance of these categories may reflect their broader semantic variability, sparse representation, and frequent dependence on article-level context rather than isolated sentence-level cues.

h. Typos. Section 4 is incorrectly titled as "Conslusions".

Thanks for pointing out this typo. We have corrected it.

i. Grammar errors. In section 2.4, "The model performance...are summarized" should be "is summarized". In section 2.3, the meaning of the term "superior identifier recognition" is unclear.

We thank the reviewer for pointing out these grammar and clarity issues. We corrected the subject–verb agreement and comprehensively revised the corresponding Results paragraphs during the repeated-run analysis. The previous phrase “superior identifier recognition” and the statement describing DeBERTa-v3

as the preferred architecture were removed. The revised manuscript now states that DeBERTa-v3 achieved the highest mean F1 for formal ethics approval identifiers, but did not significantly outperform all competing architectures after multiple-comparison correction. Model performance for the experimental-context categories is now summarized in Table 6.
